# Broadening the Agent Preference Hypothesis Through Experiencers: Eye-Tracking and EEG Evidence of Proto-Agents and Proto-Patients

**DOI:** 10.1162/OPMI.a.372

**Published:** 2026-07-17

**Authors:** Marta Sánchez-López, Arrate Isasi-Isasmendi, Balthasar Bickel, Mikel Santesteban

**Affiliations:** Department of Linguistics and Basque Studies, University of the Basque Country (UPV/EHU), Vitoria-Gasteiz, Spain; Université de Pau et des Pays de l’Adour, Bayonne, France; CNRS-IKER UMR 5478, Bayonne, France; Institute for the Interdisciplinary Study of Language Evolution (ISLE), University of Zurich, Zurich, Switzerland

**Keywords:** event roles, proto-roles, agent preference, eye-tracking, EEG

## Abstract

Despite the large number of proposed event roles, psycholinguistic evidence supports only two core knowledge categories with distinct processing correlates: agent and patient roles. This evidence aligns with the proto-role approach, which proposes only two proto-roles, proto-agent and proto-patient. Here, we investigate the processing correlates of arguments labeled as “experiencers” to determine whether they exhibit specific processing correlates, as agents and patients do, or whether they are subsumed under the proto-agent role category, sharing similar processing correlates with agents and differing from patients. We conducted both eye-tracking and EEG reading tasks, where Spanish speakers were instructed to read intransitive sentences with either agent, experiencer, or patient subjects. In eye-tracking, agents and experiencers elicited longer fixation times and regression counts than patients, in both the verb and post-verb regions. In EEG, we replicated the known N400 component associated with processing patients compared to both agents and experiencers. Crucially, no N400 effect was observed when comparing agents to experiencers. Additionally, patients revealed a power increase in the theta band compared to agents and experiencers, and a power decrease in the low-beta band. These findings replicate previous results supporting a transient preference for agents in language comprehension and suggest that experiencers align with agents in this regard, providing further evidence for the proto-role approach.

## INTRODUCTION

Event roles describe the functions that participants fulfil within an event. For example, in the sentence “Mary danced,” *Mary* is categorized as an agent, whereas in “Mary stumbled,” it is identified as a patient. In “Mary had fun,” *Mary* is categorized as an experiencer, a role distinct from agent and patient (Levin, 1993). There is extensive psycholinguistic evidence for agent and patient roles as two distinct role categories with specific processing correlates (Rissman & Majid, 2019). However, there is no such evidence for the experiencer role. Here, we investigate the processing correlates of these types of arguments to specify whether they have distinct processing correlates from agents and patients.

Many theoretical approaches have proposed a range of event roles (e.g., agent, experiencer, theme, patient, etc.) with no agreement on how many event roles there are or on their nature (Dowty, 1991; Levin & Rappaport Hovav, 2005; Newmeyer, 2010). Fillmore (1968) initially introduced a set of event roles including agentive, instrument, dative, factitive, locative, and objective. Later, he extended this list to include roles such as experiencer, source, goal, place, and time (Fillmore, 1971). Jackendoff (1990) proposed an alternative inventory comprising actor, patient, theme, goal, source, location, and benefactive, excluding the experiencer role, previously proposed. In contrast, Ramchand (2008) advocated for a smaller set with initiator, undergoer, and resultee. Dowty and Van Valin and collaborators opted for an even shorter set of event roles, proposing two: proto-agent and proto-patient (Dowty, 1991), or actor and undergoer (Van Valin, 1999, 2004; Van Valin & LaPolla, 1997).

Regarding the nature of event roles, Fillmore (1968, 1971) argued that event roles are discrete and non-decomposable categories. As such, the different categories of event roles do not share properties, since they are not decomposed into smaller features. Other authors such as Reinhart (2000, 2002) or Rozwadowska (1988, 1989), however, claimed that event roles are decomposed into semantic features. For example, the agent role is characterized by the feature of cause change and mental state/sentience, whereas the experiencer is characterized by the feature of mental state/sentience. Dowty (1991) followed this line of reasoning claiming that proto-roles are decomposed into semantic entailments: proto-agents are characterized by the entailments of causation, sentience, volition, and movement, whereas proto-patients by the entailments of change of state, incremental theme, being causally affected, and stationary. Van Valin (1999, 2004) and Van Valin and LaPolla (1997) based the macrorole approach on decomposition of individual verbs, instead on event role decomposition. He proposed that the macroroles actor and undergoer are generalizations over verb-specific roles (i.e., agent, experiencer, theme, etc.). Overall, these differences reflect the lack of agreement on the number and nature of event roles, questioning the existence of the experiencer role within the event role repertoire.

Psycholinguistic research has provided strong evidence that agent and patient roles are core knowledge categories, revealing a universal bias to distinguish both (Rissman & Majid, 2019). Numerous studies found a strong agent preference in language comprehension (Bornkessel-Schlesewsky & Schlesewsky, 2009; Frenzel et al., 2015; Gómez-Vidal et al., 2022; Isasi-Isasmendi et al., 2024), revealing an attentional preference toward agents (Gómez-Vidal et al., 2022), and a preference to transiently interpret the first unmarked noun in a sentence as an agent crosslinguistically (Bickel et al., 2015; Bisang et al., 2013; Bornkessel & Schlesewsky, 2006; Erdocia et al., 2009; Frenzel et al., 2015; Huang et al., 2013; Isasi-Isasmendi et al., 2024; Laka & Erdozia, 2012; Lamers, 2012; Sauppe et al., 2023; Vela-Plo et al., 2022). All these results align with the agent preference hypothesis which proposes a cognitive preference toward agents over patients (Bornkessel-Schlesewsky & Schlesewsky, 2009; Frenzel et al., 2015; Gómez-Vidal et al., 2022; Isasi-Isasmendi et al., 2023, 2024; Sauppe et al., 2023; Vela-Plo et al., 2022), potentially shared with non-human apes (Brocard et al., 2024, 2025; V. A. D. Wilson et al., 2022, 2024).

Gómez-Vidal et al. (2022) tested the processing of sentences with either agent or patient subjects preceding the verb. They used the Visual World Paradigm, where Spanish native speakers were instructed to listen to sentences while looking at four different images on the screen. One of the images was semantically related to the subject of the sentence, while the others were distractors semantically unrelated. When listening to the verb and the post-verb regions of the sentence, participants produced more looks to semantically related images in the agent condition than in the patient condition. They interpreted this finding as a greater magnitude of reactivation of agents compared to patients, suggesting a larger attention paid to agents compared to patients. This result reflects an attentional preference to fixate agents compared to patients, aligning with the agent preference hypothesis.

Different processing correlates between agents and patients have also been found in EEG reading tasks, particularly an N400 effect toward patient disambiguation when encountering ambiguous arguments. Haupt et al. (2008) investigated the reanalysis of arguments in verb-final transitive sentences in German. They included (1.a) agent-initial complement clauses and (1.b) patient-initial complement clauses:

(1) a. … *dass Bertram Surferinnen gratuliert hat*.
     that Bertram surfers_FEM.PL_ congratulated Aux_SG_
     “that Bertram congratulated surfers.”
    b. … *dass Bertram Surferinnen gratuliert haben.*
     that Bertram surfers_FEM.PL_ congratulated Aux_PL_
     “that surfers congratulated Bertram.”

The first noun of both sentence types (*Bertram*) was case marked ambiguously, being both agent and patient possible interpretations, until participants read the verb. Their results revealed an N400 component for disambiguation toward the patient at the verb region.

In Basque, Isasi-Isasmendi et al. (2024) investigated the previously observed agent preference in intransitive sentences, ruling out confounds from transitivity and syntactic subject vs. object asymmetries. In an EEG reading task, Native Basque speakers were asked to read intransitive sentences presented word-by-word. The arguments could be either agent or patient, with ambiguous and unambiguous case making. They found that disambiguation toward patients compared to agents elicited an N400 component over central and posterior electrodes, along with a decrease in power in the alpha and low-beta bands. They interpreted this power decrease as revision of unexpected readings and the N400 component as a recategorization of the event role category due to the agent preference hypothesis. This N400 component linked to the agent preference is also found in languages that prefer to place patients before agents, as Äiwoo (Sauppe et al., 2023), and languages where patients typically occur without case-marking, as in Hindi (Bickel et al., 2015). A comparative re-analysis of the German, Basque, and Hindi observations showed that the N400 signal can only be well-predicted by models that include an explicit agent preference, in addition to the lexical surprisal estimates that are commonly associated with this signal (Huber et al., 2024).

Furthermore, there is psycholinguistic evidence in favor of a semantic decomposition of event roles. Kako (2006) investigated whether event roles are decomposed into primitive elements. Through several experiments, participants were asked to rate proto-role entailments (Dowty, 1991) of subjects and objects using both real and nonsense verbs in English. With both verb types, participants rated subjects as more proto-agent-like than objects, and objects as more proto-patient-like than subjects. In addition, he also observed that English speakers relied more on proto-role entailments for subject and object identification than on other semantic properties, such as “can make noise”. Rissman and Lupyan (2022) also asked English speakers to induce agent and patient role categories, but from visual stimuli representing events. They found that the entailment of volition was the most reliable entailment for the identification of event roles. Using structural priming tasks during the production of Italian sentences, Vernice and Hartsuiker (2019) also found that proto-agent entailments were associated with subjects and proto-patient entailments with objects: participants produced more active structures when patients entailed more proto-patient entailments than proto-agent ones, and more passives when patients entailed more proto-agent entailments. Together, these findings provide evidence that proto-role entailments (Dowty, 1991) have a psychological reality contributing to the identification and assignment of subjects and objects.

Despite the strong and reliable evidence for agent and patient roles, there is no evidence for the experiencer role as a specific role category within the event role repertoire. The majority of previous psycholinguistic research that investigated the processing of structures with experiencer role (Bidgood et al., 2020; Bornkessel et al., 2002, 2003; Brennan & Pylkkänen, 2010; Cupples, 2002; Do & Kaiser, 2022; Ferreira, 1994; Gattei, Dickey, et al., 2015; Gattei, Tabullo, et al., 2015; Gennari & MacDonald, 2009; Manouilidou et al., 2009; Manouilidou & de Almeida, 2013; Thompson & Lee, 2009; M. Wilson & Dillon, 2022) did not query whether the experiencer role is an independent category, different from agent and patient roles (Fillmore, 1971), or whether these argument types fit into the proto-agent category (Dowty, 1991). These studies focused on the processing correlates of *frighten*-type verbs due to their syntactic property of mapping the experiencer role onto the object position (e.g., *Mary frightened Kate_EXPERIENCER_*). They found that *frighten*-type verbs involve higher processing cost than both *fear*-type verbs (e.g., *Kate_EXPERIENCER_ feared Mary*) (Do & Kaiser, 2022; M. Wilson & Dillon, 2022), and agent-patient verbs (e.g., *Kate_AGENT_ hit Mary*) (Bornkessel et al., 2002, 2003; Brennan & Pylkkänen, 2010; Ferreira, 1994; Gattei, Tabullo, et al., 2015). Interestingly, previous studies found no processing differences between *fear*-type and agent-patient verbs (Gennari & Poeppel, 2003; Katsika et al., 2012; Ma et al., 2023), with both verb types mapping experiencer and agent respectively onto the subject position.

According to the proto-role approach (Dowty, 1991), there is no category of experiencer role, since event roles are categorized in two broad categories: proto-agent and proto-patient roles. This hypothesis aligns with previous psycholinguistic evidence. Arguments with proto-agent entailments (i.e., causation, sentience, volition, and movement) fit within the proto-agent category, and arguments with proto-patient entailments (i.e., change of state, incremental theme, being causally affected, and stationary) within the proto-patient category. Based on the Argument Selection Principle (Dowty, 1991, p. 576), the argument that exhibits the greatest number of proto-agent entailments becomes the subject. This principle entails that arguments may have different degrees of membership into a proto-role. For example, in *Mary built the house*, *Mary* exhibits all proto-agent entailments, whereas in *Mary loved the house*, *Mary* only has the proto-agent entailment of sentience. Hence, experiencer-arguments are proto-agents characterized exclusively by the proto-agent entailment of sentience; so that they are not prototypical proto-agents.

Sánchez-López (2026) asked directly whether the experiencer role has similar processing correlates to agents and different correlates from patients due to their proto-agent entailment of sentience. She conducted an eye-tracking reading task where Spanish speakers were instructed to read transitive sentences with different argument structures: (i) experiencer-patient, (ii) agent-experiencer, and (iii) agent-patient. Results revealed that agent-experiencer structures involved longer fixation times than agent-patient and experiencer-patient structures at the verb and post-verb regions. This result was interpreted as evidence of the proto-role approach (Dowty, 1991): agent-experiencer structures have two arguments with proto-agent entailments causing a competition for subject assignment as both arguments are possible candidates for subjecthood.

Here, we further investigate the processing correlates of experiencer arguments by asking whether they display specific processing correlates, as agents and patients do, or whether they show similar processing correlates to agents and different ones from patients due to their proto-agent entailment, based on the proto-role approach (Dowty, 1991). These comparisons are crucial for better understanding how broad the repertoire of event roles is, and for gaining deeper insight into the mechanisms underlying the syntax-semantics interface.

In line with our previous study with transitive sentences (Sánchez-López, 2026), in Experiment 1, we conducted an eye-tracking reading task with intransitive sentences. The use of intransitive structures allowed us to directly compare the processing correlates of agents (2.a), experiencers (2.b), and patients (2.c) in the same subject position. To our knowledge, no previous eye-tracking study has investigated such event role processing differences. Next, based on previous EEG experiments on event role processing (Bickel et al., 2015; Haupt et al., 2008; Isasi-Isasmendi et al., 2024; Sauppe et al., 2023), in Experiment 2 we collected the electrophysiological responses of participants while reading the same intransitive sentences as those used in Experiment 1. The use of these two research methods with the same set of stimuli allowed us to provide a broader perspective on the processing of event roles. Furthermore, we chose Spanish as our target language because it allows minimal differentiation of the three roles without immediate consequences for surface syntax (in terms of case marking, expletive pronouns, reflexivization, word order, or agreement):

(2) a. María bailó.
     *Mary_AGENT_ danced.*
    b. María disfrutó.
     *Mary_EXPERIENCER_ had fun.*
    c. María tropezó.
     *Mary_PATIENT_ stumbled.*

Since experiencers are characterized by the proto-agent entailment of sentience, we predicted that if experiencer arguments fit into the proto-agent role (Dowty, 1991), they would have parallel processing correlates to agents and different correlates from patients at the verb position. Otherwise, if the experiencer role constitutes a specific category within the event role repertoire (Fillmore, 1971), we predicted that the experiencer would show a specific processing pattern, different from both agents and patients.

## EXPERIMENT 1

### Participants

Thirty native Spanish speakers (24 = female; age range: 18–34; mean age = 21.13; *SD* = 3.95) participated in the eye-tracking experiment for payment. All participants had normal or corrected to normal vision. This study was approved by the Ethics Committee for Research Involving Human Beings of the University of the Basque Country, and all participants gave written informed consent.

### Materials

The experiment had a repeated measures design with the Event Role condition comprising three levels: Agent, Experiencer, and Patient. Ninety-six experimental items were created, each appearing in three different sentence versions corresponding to the three conditions (see Table 1). Sentences were distributed across three counterbalanced lists following a Latin Square design; each participant only saw one of the versions of each experimental item. Experimental sentence versions differed solely in the verb used to manipulate the Event Role. The experiment included ten practice items, ninety-six experimental sentences, and ninety-six fillers. Comprehension questions were added to thirty-three percent of the items, resulting in sixty-four items with comprehension questions: thirty-two experimental items and thirty-two fillers. Comprehension questions referred to the semantic content of different parts of the sentence, and their answers were balanced: thirty-two questions had *yes* correct answer and thirty-two had *no* correct answer.

**Table 1.** Example of the 3 conditions of an experimental item in Experiments 1 and 2.

	**Event role**	**Subject Region**	**Preverb Region**	**Verb Region**	**Post-verb Region**	**Last-word Region**
(a)	Agent	La conductora	el jueves	se esforzó	durante el	examen.
(b)	Experiencer	La conductora	el jueves	se sorprendió	durante el	examen.
(c)	Patient	La conductora	el jueves	se desorientó	durante el	examen.
		*The driver*	*on Thursday*	*made an effort/was surprised/became disoriented*	*during the*	*exam.*

Sixteen different verbs per condition were selected to create the different versions of experimental items (each verb was repeated six times to get ninety-six experimental items). Verbs were selected based on verb classifications found in *Gramática descriptiva de la lengua española* (Descriptive grammar of the Spanish language) (Bosque et al., 1999). For each condition, eight verbs had the *se* morpheme and eight without *se*, with no difference in meaning, used simultaneously within items. The length (in number of letters in the conjugated form used in the experimental sentences) and the frequency of verbs (in the infinitive form due to the lack of frequency of some verbs in their conjugated form) was controlled using the SUBTLEX-ESP corpus (Cuetos et al., 2011). Paired *t*-test analyses revealed no differences in either length or frequency among conditions (all posterior differences have a 95% credible interval closely around zero; see Appendix I (available in the OSF repository indicated in the Data Availability Statement section).

Experimental items were previously normed for naturalness. Forty-four native speakers of Spanish filled an online sentence naturalness questionnaire in PCIbex (Zehr & Schwarz, 2022), and they had to rate the naturalness of sentences on a 5-point Likert scale. All conditions were rated above 3.5 (Agent: mean = 3.92, *SD* = 1.16; Experiencer: mean = 3.74, *SD* = 1.23; Patient: mean = 3.80, *SD* = 1.23). We found acceptability rating differences between Agent and Experiencer condition ($\hat{\beta }$_Agent-Experiencer_ = −0.035, *SE* = 0.012, 95% CrI = [−0.58, −0.12]), but not between Agent and Patient ($\hat{\beta }$_Agent-Patient_ = −0.019, *SE* = 0.013, 95% CrI = [−0.45, 0.06]), or between Experiencer and Patient ($\hat{\beta }$_Experiencer-Patient_ = 0.015, *SE* = 0.011, 95% CrI = [−0.07, 0.37]).

Additionally, to control the cloze predictability of experimental sentences as a potential confound of N400 signals (Frank et al., 2015; Michaelov et al., 2024), their surprisal values were calculated using a pretrained GPT-2 model from HuggingFace and a library provided by Remo Nitschke (University of Zurich; https://github.com/remo-help/surprisal_from_llm). We obtained the surprisal values of each word of our experimental sentences as the sentence unfolds. We compared the surprisal values of experimental sentences across the three conditions and found no notable differences among them either in the verb region ($\hat{\beta }$_Agent-Patient_ = −0.16, *SE* = 0.46, 95% CrI = [−1.05, 0.74]; $\hat{\beta }$_Agent-Experiencer_ = 0.41, *SE* = 0.46, 95% CrI = [−0.50, 1.32]; $\hat{\beta }$_Experiencer-Patient_ = −0.55, *SE* = 0.45, 95% CrI = [−1.44, 0.33]), or in the post-verb region ($\hat{\beta }$_Agent-Patient_ = −0.22, *SE* = 0.32, 95% CrI = [−0.85, 0.39]; $\hat{\beta }$_Agent-Experiencer_ = 0.27, *SE* = 0.32, 95% CrI = [−0.36, 0.90]; $\hat{\beta }$_Experiencer-Patient_ = −0.49, *SE* = 0.32, 95% CrI = [−1.11, 0.14]).

### Procedure

Eye-movements were recorded with an EyeLink 1000 Plus desktop eye-tracker sampling at 1000 Hz. Calibration and tracking were performed on each participant’s dominant eye. Participants were seated 55.5 cm from the camera, with the display screen positioned 92 cm away. Stimuli were displayed in black on a light grey background (RGB values: 217, 217, 217), appearing in a single line in Courier New Bold font type at size 26. Stimuli were positioned at screen coordinates (60, 540) on a monitor with a resolution of 1920 × 1080 pixels.

The experiment began with a calibration and validation phase using a 9-points grid for both processes. In cases where participants wore contact lenses and accurate calibration at the screen corners was not feasible, a 5-points calibration was used instead. Since stimuli were presented in a single line at the vertical centre of the screen, calibrating the corner points was not critical. Participants were instructed to read sentences and to answer comprehension questions after some of the sentences. Each trial was initiated automatically after participants fixated on a left-central fixation point, corresponding to the position where the first letter of the sentence was to appear. Participants had 3000 ms to fixate this point and to maintain their sight at the fixation point during 500 ms for the stimulus to appear. If gaze was not detected, the trial was recycled and the calibration and validation procedure was repeated.

Participants responded using a button-box: the left button for *yes*-type responses, the right button for *no*-type responses, and the middle button to proceed to the next stimulus. Trials were presented in five blocks of 38/39 trials, with four pauses for participants to rest. The entire session lasted around 60 minutes.

### Data Analysis

The dependent variables in this study included Gaze Duration, Regression Path Duration, Re-reading Duration, Regression Out Count, Regression In Count, and Total Duration. Eye-tracking measures are categorized into three temporal stages: early, intermediate, and late measures.

Early measures are associated with word recognition and lexical process and late measures with argument structure integration (Conklin et al., 2018). The Gaze Duration variable, an early measure, captures the time spent fixating the target region during the first pass, before leaving the region to look at any other region to the right or to the left. Re-reading Duration, and Regression Path Duration are intermediate measures. The Regression Path Duration variable reflects the time participants have fixated on the target region before having fixated any region on the right, but it may include regressions from the left. The Re-reading Duration is calculated as the difference between Regression Path Duration and Gaze Duration. The Regression Out Count variable measures the number of regressions from the target region to a region on the left before any rightward movement while the Regression In Count variable tracks entries into the target region from the right. The Total Duration variable, a late measure, includes the total time participants have been looking at the target region, including re-reading times from both sides (Conklin et al., 2018; SR Research, 2020). For an exemplification of the eye-tracking measures, see Appendix V.

To our knowledge, no prior eye-tracking reading study has directly investigated event role specificities, so we did not have a specific connection between eye-tracking measures and the assignment of event roles. Hence, we decided to include standard measures used in previous studies (e.g., Gattei et al., 2018; Katsika et al., 2012; Ma et al., 2023). We hypothesized that early measures, such as Gaze Duration, would reflect more automatic stages of processing, including stimulus recognition and lexical access (Conklin et al., 2018). Hence, results found in this measure might reflect differences in the lexical access of verbs, showing semantic differences between them that would not be related with differences in the assignment of event roles. In contrast, we expected intermediate and late measures to index integrative processes (Conklin et al., 2018; Gattei et al., 2018), reflecting mechanisms underlying the syntax-semantics interface. Therefore, we predicted that differences in these intermediate and late eye-tracking measures would be related to event role assignment based on verb information.

Experimental items were split into five regions: subject region, preverb region, verb region, post-verb region, and last-word region (see Table 1). The last-word region was included to avoid wrap-up effects (Warren et al., 2009). We expected to find effects in the verb and the post-verb regions, since, at these regions, participants would be able to confirm the event role assigned to the subject or to reassign a new role if the verb disconfirms the initial assignment.

Fixations shorter than 80 ms and longer than 800 ms were removed using DataViewer software (SR Research Ltd., 2022). No participant was excluded due to accuracy criteria, since all participants showed accuracy rates over 80% in the comprehension questions.

Data analysis was conducted in R (R Core Team, 2025), using Bayesian Mixed-Effects Models with *brms* function (Bürkner, 2017). For Gaze Duration, Regression Path Duration, Re-reading Duration and Total Duration measures, we created Bayesian Mixed-Effects Models with a Shifted-lognormal likelihood. For Regression Out Count and Regression In Count variables a Poisson likelihood was used.

We specified uninformative normally distributed priors for intercepts and slopes to reflect plausible uncertainty while allowing the data to drive estimates (Kruschke, 2015; Nicenboim et al., 2025). To specify the prior distributions of the parameters of models with a Shifted log-normal likelihood, we chose normal distributions for the intercept (*μ* = 5, *σ* = 1 for Gaze Duration and Regression Path Duration; *μ* = 6, *σ* = 1 for Re-reading Duration; and *μ* = 6, *σ* = 1 for Total Durations) and for the slopes (*μ* = 0, *σ* = 2 for all measures). For models with a Poisson likelihood, we opted for normal distributions for the intercept (*μ* = −1, *σ* = 2 for Regression In Count; *μ* = −3, *σ* = 2 for Regression Out Count) and slopes (*μ* = 0, *σ* = 2) parameters due to convergence issues with the conjugate Gamma distribution.

To account for sentence predictability, we included surprisal values as a predictor in the models (Huber et al., 2024). This ensured that any observed effects were not solely driven by difference in lexical predictability. We compared models using leave-one-out cross-validation (loo-cv) (McElreath, 2020; Vehtari et al., 2017), with the stacking technique for model weighting (Yao et al., 2018).

For each region and dependent variable, we fitted and compared four models: a null model without predictors, a model with only surprisal, a model with only Event Role predictor, and a model with surprisal and Event Role predictors. The stacking weights derived from loo-cv reflect each model’s relative performance, based on how well it predicts left-out observations (Vehtari et al., 2017).

We reported the marginal comparisons from the highest-weight model for each region and variable. These comparisons estimate the average difference in the predicted outcome between levels, averaging over the random effect structure (Arel-Bundock et al., 2024). When the highest-weight model was the null model, we did not report anything. Model comparisons are shown in Appendix II.

### Results

#### Surprisal Values.

In this section, we briefly discuss the effect of lexical surprisal values. All these models revealed a positive surprisal effect showing that participants made higher fixation times, and more regression counts with higher surprisal values than with lower surprisal values. This effect appeared in the preverb region in Regression Path Duration ($\hat{\beta }$_Surprisal_ = 0.04, 95% CrI = [0.01, 0.06]), in the verb region in Gaze Duration ($\hat{\beta }$_Surprisal_ = 0.07, 95% CrI = [0.04, 0.10]) ($\hat{\beta }$_Surprisal_ = 15.1, 95% CrI = [15.1, 49.1]), Regression Path Duration ($\hat{\beta }$_Surprisal_ = 0.08, 95% CrI = [0.05, 0.12]), and Total Duration ($\hat{\beta }$_Surprisal_ = 0.07, 95% CrI = [0.03, 0.10]), in the post-verb region in Gaze Duration ($\hat{\beta }$_Surprisal_ = 0.05, 95% CrI = [0.02, 0.09]), Re-reading Duration ($\hat{\beta }$_Surprisal_ = 0.08, 95% CrI = [0.02, 0.15]), Regression In Count ($\hat{\beta }$_Surprisal_ = 0.17, 95% CrI = [0.09, 0.25]), and Total Duration ($\hat{\beta }$_Surprisal_ = 0.09, 95% CrI = [0.07, 0.12]).

#### Eye-Tracking Measures.

**Figure 1.**
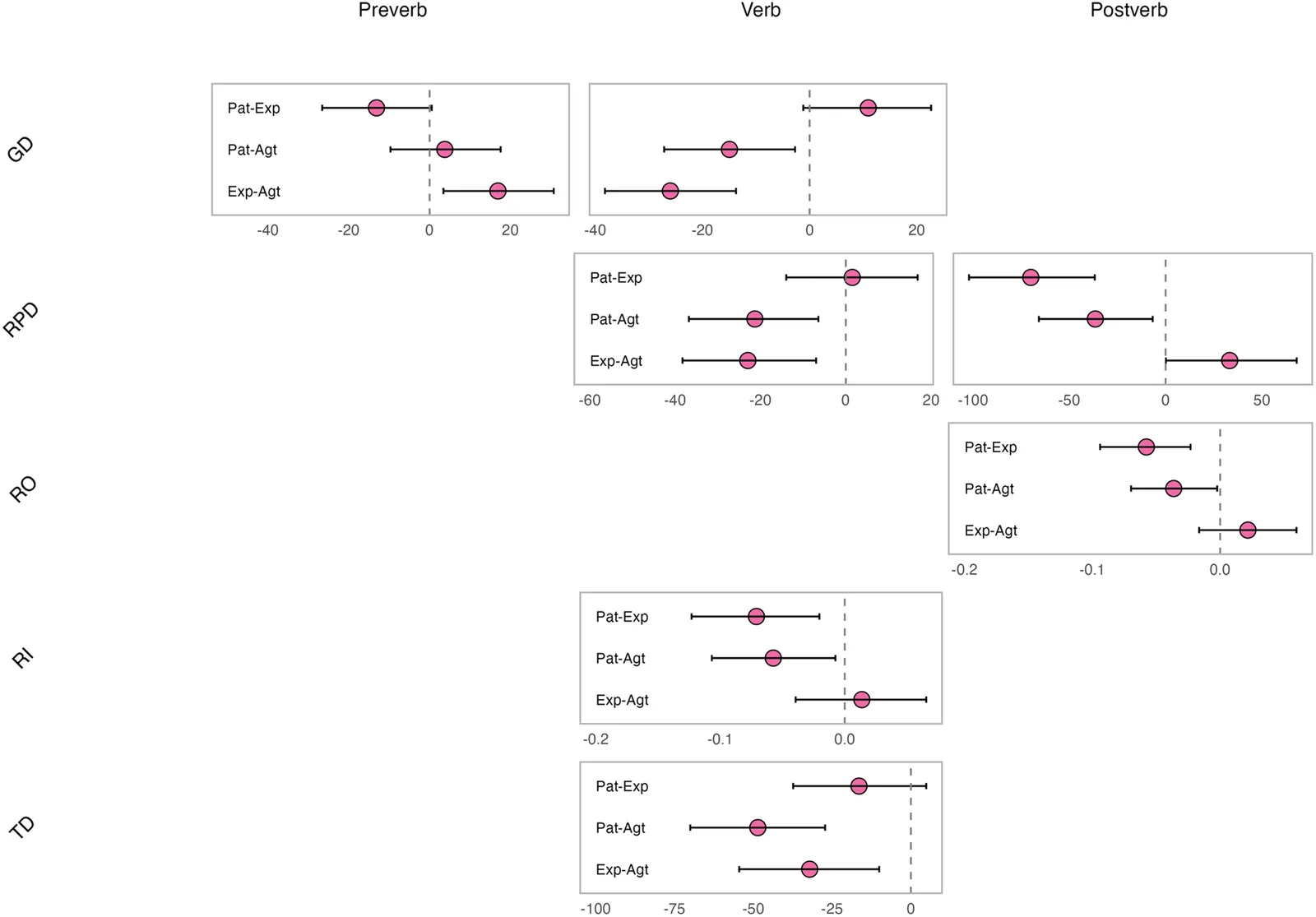
Marginal estimates of pairwise condition contrasts for eye-tracking measures across sentence regions. Each row corresponds to a different eye-tracking measure: Gaze Duration (GD), Regression Path Duration (RPD), Regression Out Count (RO), Regression In Count (RI), and Total Duration (TD). Each column represents a sentence region. For each region and measure, estimated differences between conditions (Pat-Exp, Pat-Agt, Exp-Agt) are shown as dots, with horizontal lines indicating 95% credible intervals. The *x*-axis is in milliseconds (ms), except for RI and RO that is in counts.

Figure 1 displays marginal estimates of pairwise condition contrasts for eye-tracking measures across sentence regions. Marginal comparisons revealed that the agent condition involved longer fixation times than the patient condition in the verb region in Gaze Duration ($\hat{\beta }$_Agent-Patient_ = −15, CrI = [−27, 14, −2.71]), Regression Path Duration ($\hat{\beta }$_Agent-Patient_ = −22.97, CrI = [−38.3, −6.96]), Regression In Count ($\hat{\beta }$_Agent-Patient_ = −0.0574, CrI = [−0.1068, −0.00749]), and Total Duration ($\hat{\beta }$_Agent-Patient_ = −48.6, CrI = [−70, −27.24]). We found this same effect in the post-verb region in Regression Path Duration ($\hat{\beta }$_Agent-Patient_ = −36.5, CrI = [−65.783, −6.7]), and Regression Out Count ($\hat{\beta }$_Agent-Patient_ = −0.0364, CrI = [−0.0697, −0.00231]).

The experiencer condition showed higher fixation counts than the patient condition in the verb region in Regression In Count ($\hat{\beta }$_Experiencer-Patient_ = −0.0710, CrI = [−0.1231, −0.02037]). This effect appears less robustly in Total Duration ($\hat{\beta }$_Experiencer-Patient_ = −16.5, CrI = [−37.4, 4.87]). Although the credible interval overlaps 0, the direction of the effect and the credible interval indicate a higher probability that the experiencer condition elicited longer total fixation times than the patient condition. In the post-verb region, the effect continues in Regression Path Duration ($\hat{\beta }$_Experiencer-Patient_ = −70, CrI = [−102.082, −36.8]), and Regression Out Count ($\hat{\beta }$_Experiencer-Patient_ = −0.0578, CrI = [−0.0940, −0.02320]).

When comparing agent and experiencer conditions, the effect and its direction are not as clear as in the other two comparisons. In the preverb region, in Gaze Duration, the experiencer condition showed longer fixation times than the agent condition ($\hat{\beta }$_Agent-Experiencer_ = 16.91, CrI = [3.42, 30.734]). In the verb region, the direction of the effect shifted revealing longer fixation times for the agent compared to the experiencer condition in Gaze Duration ($\hat{\beta }$_Agent-Experiencer_ = −26, CrI = [−38.19, −13.73]), Regression Path Duration ($\hat{\beta }$_Agent-Experiencer_ = −21.32, CrI = [−36.8, −6.43]), and Total Duration ($\hat{\beta }$_Agent-Experiencer_ = −32.1, CrI = [−54.5, −10.05]). In the post-verb region, in Regression Path Duration, the direction of the effect changed again showing longer fixation times for the experiencer than for the agent condition ($\hat{\beta }$_Agent-Experiencer_ = 33.3, CrI = [0.159, 68.1]). Differences between both disappear in the verb region in Regression In Count ($\hat{\beta }$_Agent-Experiencer_ = 0.0138, CrI = [−0.0394, 0.06551]), and in the post-verb region in Regression Out Count ($\hat{\beta }$_Agent-Experiencer_ = −0.0216, CrI = [−0.0597, 0.01649]).

### Discussion

In the eye-tracking reading experiment, several measures revealed a consistent pattern: agents and experiencers involved longer fixation times and regression counts than patients, though the differences between agents and experiencers varied depending on the measure and region.

Although larger fixation times and higher regression counts have typically been associated with higher processing costs in eye-tracking (Conklin et al., 2018), more fixations have also been related to an attentional preference toward a stimulus (Cohn & Paczynski, 2013; Galazka & Nyström, 2016; Gómez-Vidal et al., 2022; Isasi-Isasmendi et al., 2023). In the present eye-tracking reading experiment, our findings support the latter interpretation: we found that agent and experiencer conditions elicited longer fixation times than the patient condition in the verb and post-verb region, as well as more regressions to the verb. These results are consistent with previous evidence that intransitive verbs with agents led to longer reaction times than those with patients in acceptability tasks (Martinez de la Hidalga et al., 2019), and to longer regression counts in eye-tracking reading tasks (Zeyrek & Acartürk, 2014). Furthermore, they align with prior evidence showing an attentional preference toward agents, both in language processing (Gómez-Vidal et al., 2022) and in event processing (Cohn & Paczynski, 2013; Isasi-Isasmendi et al., 2023).

A detailed examination of the eye-movement patterns revealed that the experiencer condition involved longer gaze durations than agent and patient conditions in the preverbal region, but in the verb region, the experiencer condition showed shorter gaze durations than both agent and patient conditions. Since Gaze Duration is associated with early word recognition and lexical access processes (Conklin et al., 2018), this pattern suggests that the lexical access to verbs selecting prototypical event roles (i.e., agents and patients) differs from that to verbs selecting less prototypical event roles (i.e., experiencers). The fact that this effect appeared first in the preverbal region suggests a parafoveal effect, as participants had not seen yet the verb region (Schotter & Dillon, 2025). This initial relation between agents and patients in comparison to experiencers may be related to the prototypicality of the arguments. Agents and patients are the two prototypical event roles, whereas experiencers are less prototypical (Dowty, 1991; Levin & Rappaport Hovav, 2005; Li, 2020; Van Valin, 2004). Based on the proto-role approach and on the Argument Selection Principle (Dowty, 1991), both agents and experiencers are arguments characterized by proto-agent entailments, but experiencers are less prototypical than agents because they only exhibit the entailment of sentience.

The next measure, Regression Path Duration, is an intermediate measure, between lexical access and integration processes (Conklin et al., 2018). We found that the agent condition involved larger regression path durations than both patient and experiencer conditions in the verb region. However, in the postverbal region, this pattern shifted and now both agent and experiencer conditions showed longer regression path durations than the patient condition. The delayed increase for experiencers may reflect a later categorization as proto-agents, due to their unique proto-agent entailment, the one of sentience (Dowty, 1991). This later activation of experiencers converges with Gómez-Vidal’s (2024) Visual World Paradigm results. In her PhD thesis, she statistically compared her data from different experiments, getting a comparison of the activation of agent, experiencer, and patient subjects in the post-verb region. She found that experiencers and agents in intransitive sentences elicited a larger proportion of fixations than patients, without differences between experiencers and agents. However, regarding the time course of the proportion of fixations to pictures semantically related to the subject (as compared to unrelated ones), both agents and patients involved first an increase of fixations, followed by a decrease, and ending with an increase, whereas experiencers displayed first a decrease, next an increase, and finally a decrease. These results align with our eye-tracking results showing that experiencers behave like agents but involve an activation delay.

In Regression In Count, Regression Out Count, and Total Duration measures, all late measures linked to integration processes (Conklin et al., 2018), agent and experiencer conditions displayed larger regression counts and fixation times than the patient condition. Altogether, these results suggest that both agents and experiencers received greater attentional focus, evidenced by longer fixation durations and regression counts than patients, with experiencers showing a delayed attentional effect consistent with their non-prototypical status as proto-agents.

In Experiment 2, we recorded the EEG responses elicited while different participants read the same materials as in Experiment 1. This methodology allowed us to measure the transient interpretation of ambiguous arguments and their recategorization. In Experiment 1, we obtained results related to the attentional preference of event roles. By contrast, in Experiment 2, we aimed to report the N400 component previously found in the literature associated with a recategorization toward patients (Bickel et al., 2015; Haupt et al., 2008; Isasi-Isasmendi et al., 2024; Sauppe et al., 2023), as well as the decrease in power in alpha and low-beta bands (Isasi-Isasmendi et al., 2024), to see whether this recategorization also emerges toward experiencers or not.

A power decrease in alpha band has been related with a release of inhibition of the patient category due to the activation of the agent category (Isasi-Isasmendi et al., 2024). A power decrease in low-beta band has been proposed to appear with changes in the current cognitive set activated (Isasi-Isasmendi et al., 2024; Lewis & Bastiaansen, 2015; Meyer, 2018), reflecting a power decrease with verbs selecting patient role (Isasi-Isasmendi et al., 2024). Furthermore, we also analyzed the theta band, where a power increase has been proposed to be related with unexpected words (Klimesch, 1999; Mellem et al., 2013; Rommers et al., 2017; Weiss et al., 2000). Hence, the recategorization effect in Experiment 2 should also be reflected in a theta power increase, and in an alpha and low-beta power decrease.

## EXPERIMENT 2

### Participants

Thirty native speakers of Spanish participated in the EEG experiment for payment. They were all right-handed and between 18–34 years old (22 = female, mean age = 20.43; *SD* = 2.66). This study was approved by the Ethics Committee for Research Involving Human Beings of the University of the Basque Country and all participants gave written informed consent.

### Materials

We used the same material as in Experiment 1.

### Procedure

Stimuli were presented on a PC using E-prime 3.0 (version 3.0.3.80) (W. Schneider et al., 2012). The sentences were displayed word by word at the centre of the screen in Arial Bold font type, size 40. For verbs with *se* (e.g., *se despertó*), both words were shown together. Each word was presented for 450 ms, followed by a 350 ms inter-stimulus interval. A fixation cross lasting 1000 ms indicated the beginning of each sentence trial. As in the eye-tracking experiment, participants saw comprehension questions after thirty-three percent of the sentences. They pressed 1 on the keyboard to respond *yes*, and 2 to respond *no*.

All 192 sentences were randomly distributed in 5 blocks. Prior to the sentence comprehension task, participants received instructions about the EEG procedure. The experimental session was preceded by ten practice items. EEG resting-state activity was recorded both before and after the sentence comprehension task to extract individual peak alpha frequencies for specifying individual frequency bands. The entire experiment lasted about 120 minutes, including electrode-cap application and removal.

Electrophysiological activity was recorded using 32 active electrodes secured in an elastic cap (Acticap System, Brain Products), positioned according to the extended international 10–20 system. All recording were referenced online to the right mastoid electrode and re-referenced off-line to the linked mastoids. Vertical and horizontal eye movements and blinks were monitored using two electrodes placed below and to the right of the right eye. Electrode impedances were kept below 5 Ω. The electrical signals were digitalized online at a sampling rate of 500 Hz using a Brain Vision amplifier system.

### Data Processing and Analyses

Accuracy data revealed that all participants showed accuracy rates over 80% in the comprehension questions; hence, no participant was excluded due to accuracy criteria.

EEG data were preprocessed using EEGLAB (Delorme & Makeig, 2004), FieldTrip (Oostenveld et al., 2011) and R (R Core Team, 2025). Continuous EEG signals were band-pass filtered between 0.1 and 30 Hz, down-sampled to 250 Hz, and re-referenced offline to the average of the left and right mastoids. Artifactual channels were automatically excluded if their kurtosis or probability deviated by more than five standard deviations from the mean of all channels. Independent components were computed on a 1 Hz high-pass filtered copy of the data.

Artifactual independent components were identified using the SASICA algorithm (Semi-Automated Selection of Independent Components of the electroencephalogram for Artifact correction) (Chaumon et al., 2015; Nolan et al., 2010) and excluded if they were spatially limited to single electrodes or showed high correlation with the activity of EOG electrodes. Previously rejected channels were spherically interpolated after removal of the artifactual components from the EEG data.

For the ERP analyses, EEG data were epoched from −200 to 900 ms relative to the critical word (the verb). For event-related power analyses, epochs ranged from −1500 to 1200 ms relative to the critical word. In both analyses, epoch-level artifact detection and trial-wise interpolation were performed to clean the data of temporary artifacts, using the Trial-By-Trial (TBT) interpolation method. For time-frequency analysis, single-trial data were transformed into time-frequency representations in 0.5 Hz steps using wavelet decomposition via the multi-taper method convolution with Hanning-tapered time-windows. Each window had a length of 3 cycles and advanced in steps of 50 ms. Power spectra were transformed into decibel (dB) values relative to the median power in a baseline period of −800 −500 ms before the critical word, allowing for direct comparison across frequencies (Cohen, 2014). To reduce data dimensionality, power was then averaged within predefined frequency bands.

Frequency bands were defined for each participant (Klimesch, 1999) according to their individual peak alpha frequency (IAF), following the method proposed by Corcoran et al. (2018), which estimates IAFs from resting-state EEG data. The theta band was set from IAF-6 Hz to IAF-4 Hz, alpha band from IAF-4 Hz to IAF+2 Hz (Klimesch, 1999), and the lower beta band from IAF+2 Hz to IAF+10 Hz (Bice et al., 2020). We selected these three frequency bands based on the study of Isasi-Isasmendi et al. (2024) investigating the agent preference with intransitive structures.

Statistical analyses were conducted in R using the packages *brms* (Bürkner, 2017) and *mgcv* (Wood, 2004, 2011) for modelling, and the *ggplot2* (Wickham et al., 2007) and *itsadug* packages (van Rij et al., 2020) for plotting. We analyzed the topographies of event-related potentials and event-related power changes using single-trial generalized additive mixed models (GAMMs). These models extend generalized linear models by enabling the modelling of non-linear relations between variables. In our analyses, GAMMs were used to model amplitude or power differences between experimental conditions as a function of electrode position on the scalp, represented by *x* and *y* coordinates. For event-related potentials, we used Bayesian GAMMs. However, for event-related power changes, we employed Frequentist GAMMs, as the Bayesian models faced exceedingly high computing times due to the large data volume.

For ERP analyses, the mean amplitude was modelled within a 300–500 ms time-window after the critical word onset. An additional analysis was conducted for a later window, 600–800 ms time-window after the critical word. These time-windows were selected based on prior literature (Aurnhammer et al., 2021; Bornkessel et al., 2003; Frenzel et al., 2015; Isasi-Isasmendi et al., 2024; Sauppe et al., 2023) and supported by a supplementary time-course analysis (see Appendix III). The statistical modelling approach was identical for both time-windows. The amplitude during a baseline period (averaged from −100 to 0 ms before critical word; Alday, 2019) was included as a parametric predictor. To account for potential non-linear relations between variables, a smooth term was added to model amplitude differences between conditions across the *x* and *y* scalp coordinates. Random slopes for electrode coordinates were included by participant and by item.

The statistical modelling for time-frequency analyses (TFA) closely mirrored that used for the ERPs, with two differences: the response variable was power deviations from the baselines in decibels per trial. Models were fitted separately for each frequency band. The time-windows analysed were the same as in the ERP analyses: 300–500 ms post-critical word onset for the early window, and 600–800 ms for the late time-window.

As in Experiment 1, we compared Bayesian models using leave-one-out cross-validation (McElreath, 2020; Vehtari et al., 2017), applying the stacking technique for model weighting (Yao et al., 2018). Each model comparison involved four versions: a null model without predictors, a model including only the Event Role predictor, a model including only the predictor of surprisal values, and a model including both Event Role and surprisal value predictors. For Frequentist models, we used the Akaike information criterion (AIC) for model comparison (Akaike, 1974). See Appendix VI for both ERP and TFA model comparison.

For both ERP and event-related power models, statistical significance was assessed by inspecting non-overlapping confidence intervals in the (smooth) difference surfaces. Because GAMMs model non-linear effects that cannot be captured by a single summary statistic, we followed standard procedure (Wood, 2004) and report effect sizes graphically, as model-fitted response values. Effects are reported in terms of 95% intervals (credible intervals in Bayesian models, confidence intervals in Frequentist models) around the fitted differences between critical conditions (i.e., Δ*μV* and ΔdB) visualized across the spatially smoothed electrode array.

### Results

**Figure 2.**
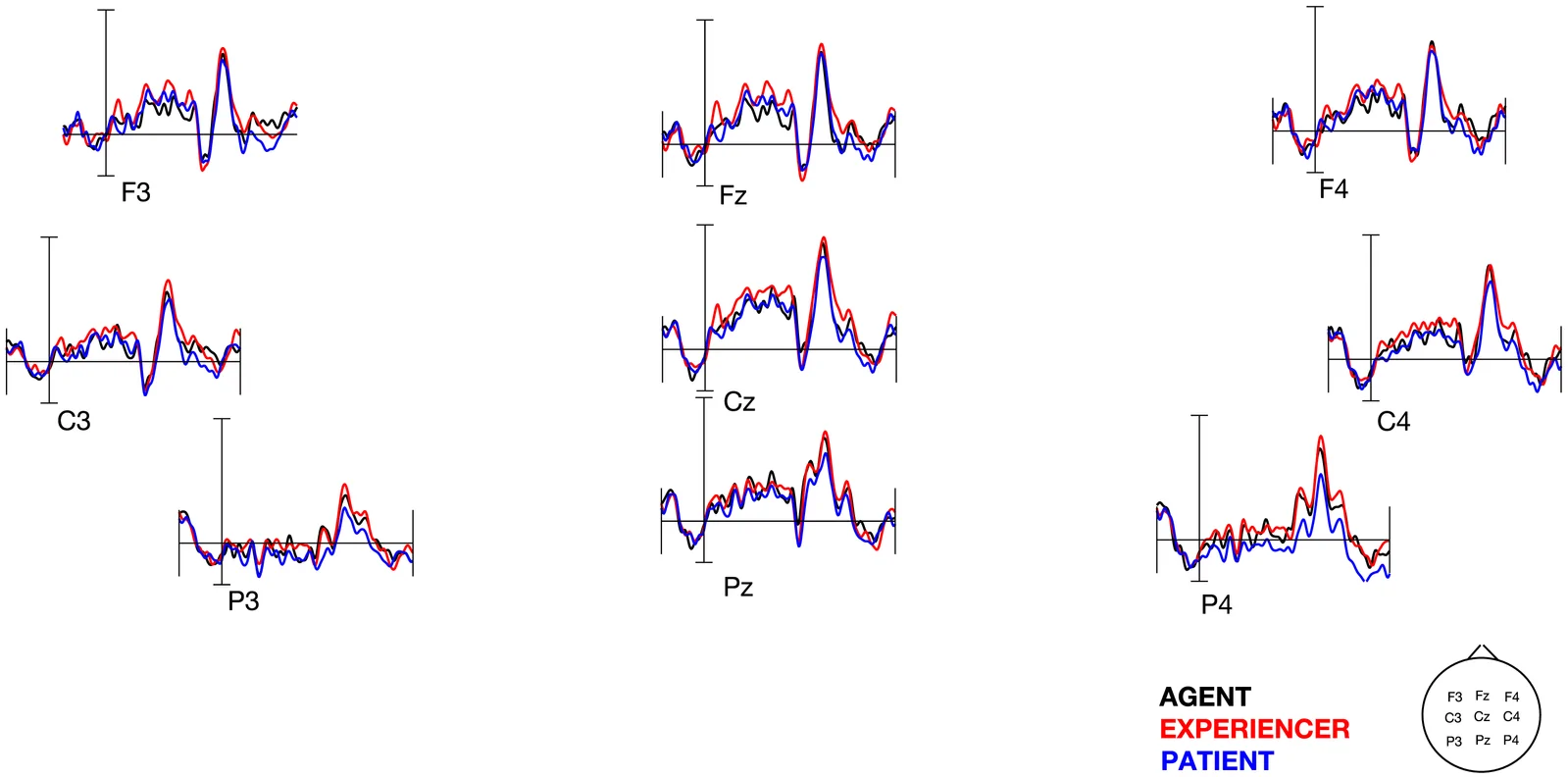
Grand average event-related potentials at the verb region. For visual clarity, the nine electrodes displayed (F3, Fz, F4, etc.) are representative of their respective scalp regions. F3, Fz, and F4 electrodes are the most anterior frontal electrodes in this figure, and they indicate the front of the head. A complete montage of all analyzed electrodes is available in the Appendix IV.

**Figure 3.**
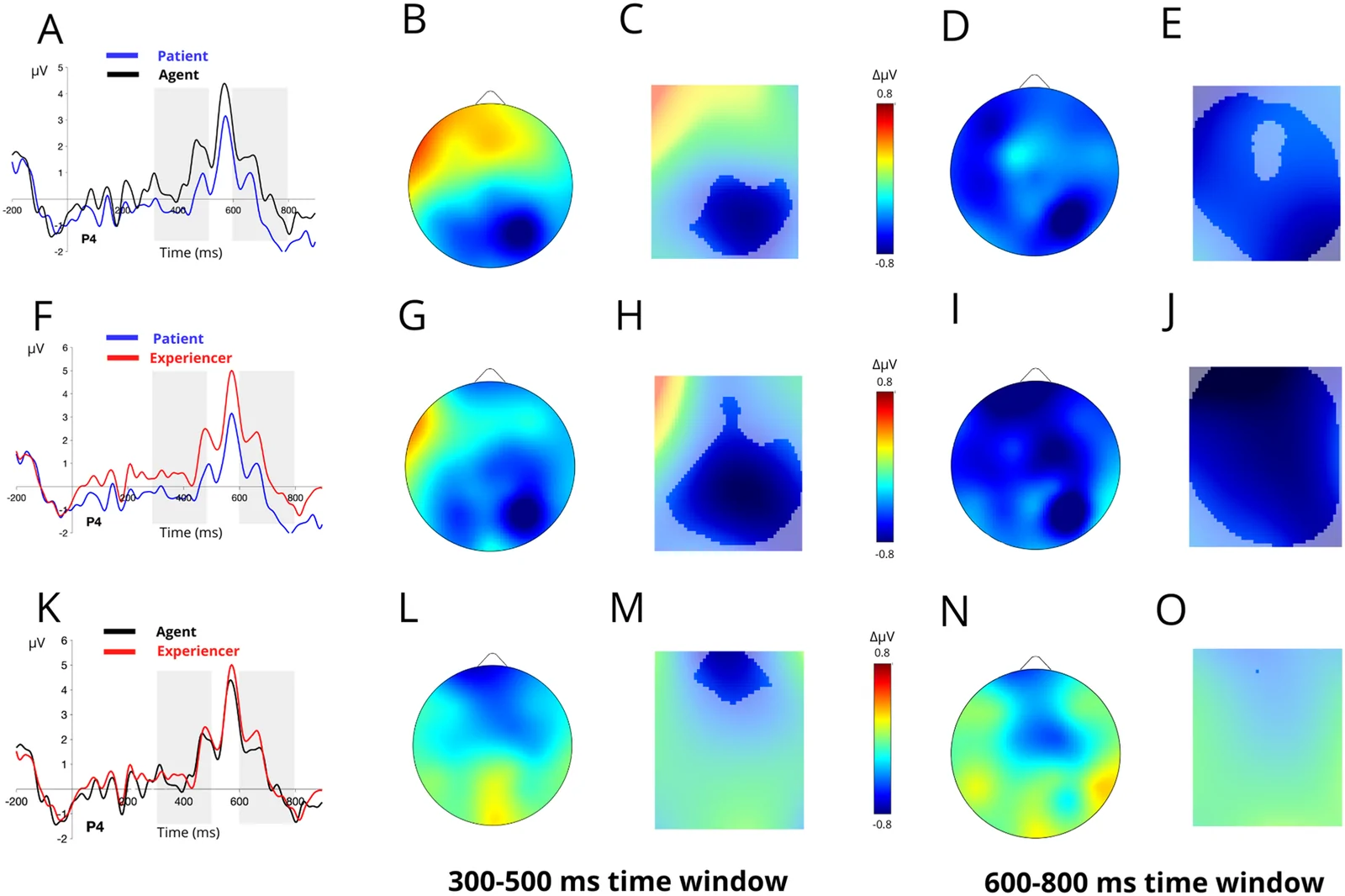
Pairwise comparisons of ERP differences between conditions at the verb region. The first row (Figure 3A, B, C, D, E) shows the contrast between Patient and Agent conditions (Δ*μV* = *μV*(Patient) − *μV*(Agent)). The second row (Figure 3F, G, H, I, J) compares Patient and Experiencer conditions (Δ*μV* = *μV*(Patient) − *μV*(Experiencer)). The third row (Figure 3K, L, M, N, O) shows the comparison between Agent and Experiencer conditions (Δ*μV* = *μV*(Agent) − *μV*(Experiencer)). Within each row, left figures (Figure 3A, F, K) display grand mean ERP time courses at the P4 electrode. Figures 3B, G, L show the topographical distribution of the grand mean differences between conditions across the scalp for the 300–500 ms time-window and Figures 3D, I, N for the 600–800 ms time-window. Figures 3C, H, M display GAMM-derived surface maps of condition differences from the highest-weighted model during the 300–500 ms time-window and Figures 3E, J, O during the 600–800 ms time-window. In the GAMM plots, bright areas indicate differences whose 95% credible interval excludes zero.

**Figure 4.**
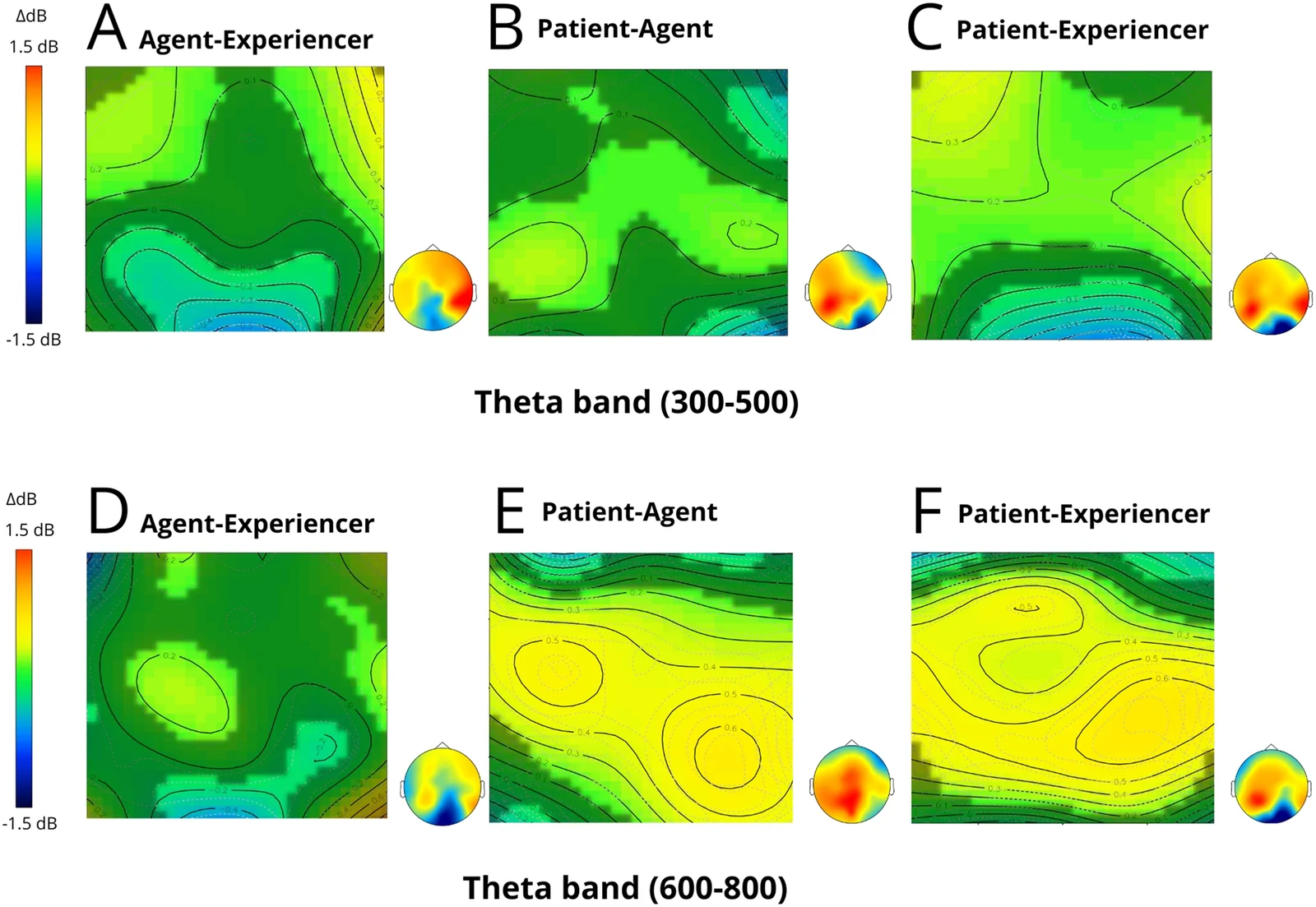
Comparison of power differences in individually defined theta band in the 300–500 ms time-window (A, B, C) and in the 600–800 ms time-window (D, E, F) after critical word presentation. Large panels show estimated (GAMM) difference surfaces of the topographic distribution of differences between conditions. Adjacent small scalp plots show the grand-average at the time-frequency point of maximal difference between conditions within each band and time-window. Non-shaded (bright) areas indicate significant differences.

**Figure 5.**
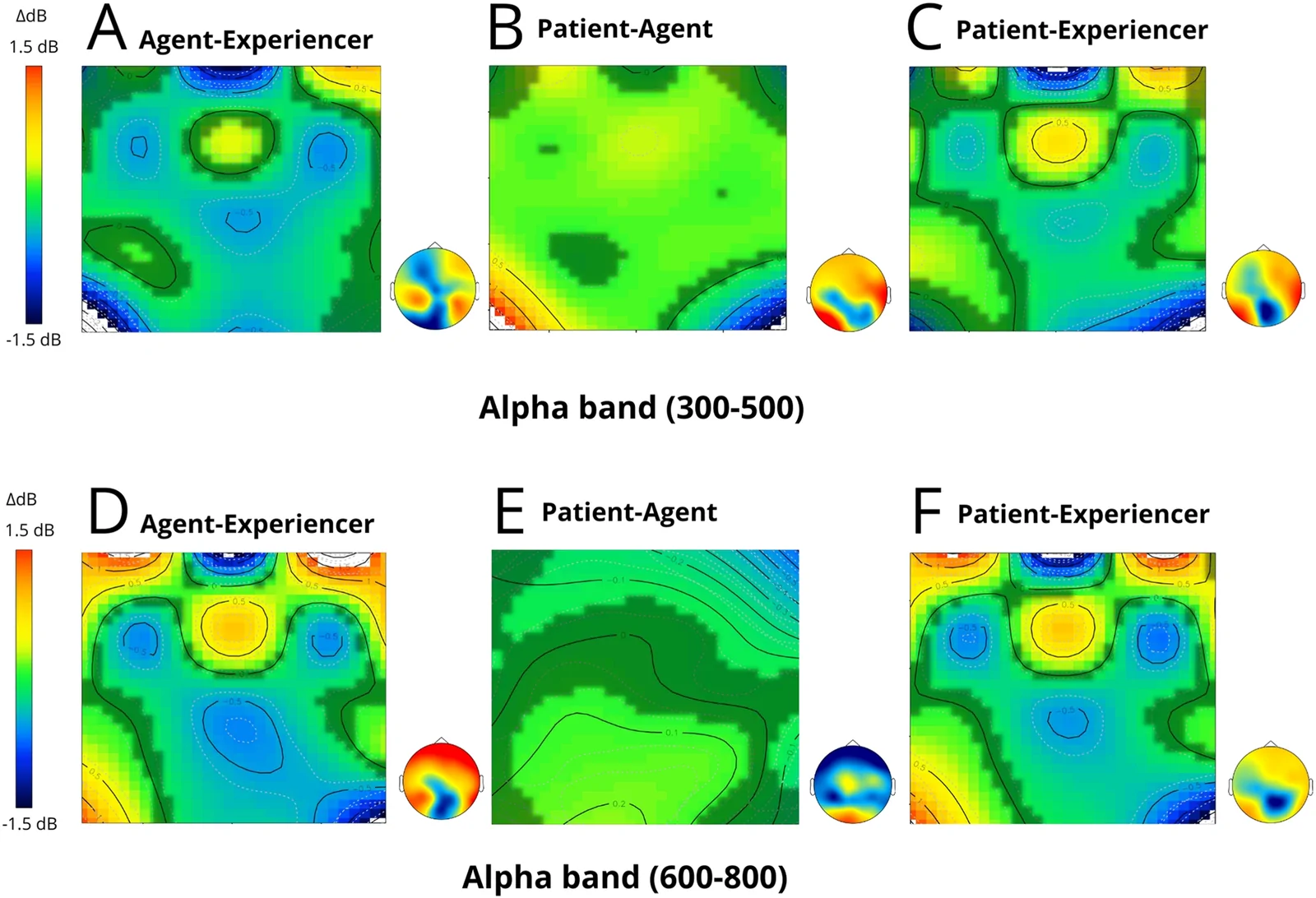
Comparison of power differences in individually defined alpha band in the 300–500 ms time-window (A, B, C) and in the 600–800 ms time-window (D, E, F) after critical word presentation. Large panels show estimated (GAMM) difference surfaces of the topographic distribution of differences between conditions. Adjacent small scalp plots show the grand-average at the time-frequency point of maximal difference between conditions within each band and time-window. Non-shaded (bright) areas indicate significant differences.

**Figure 6.**
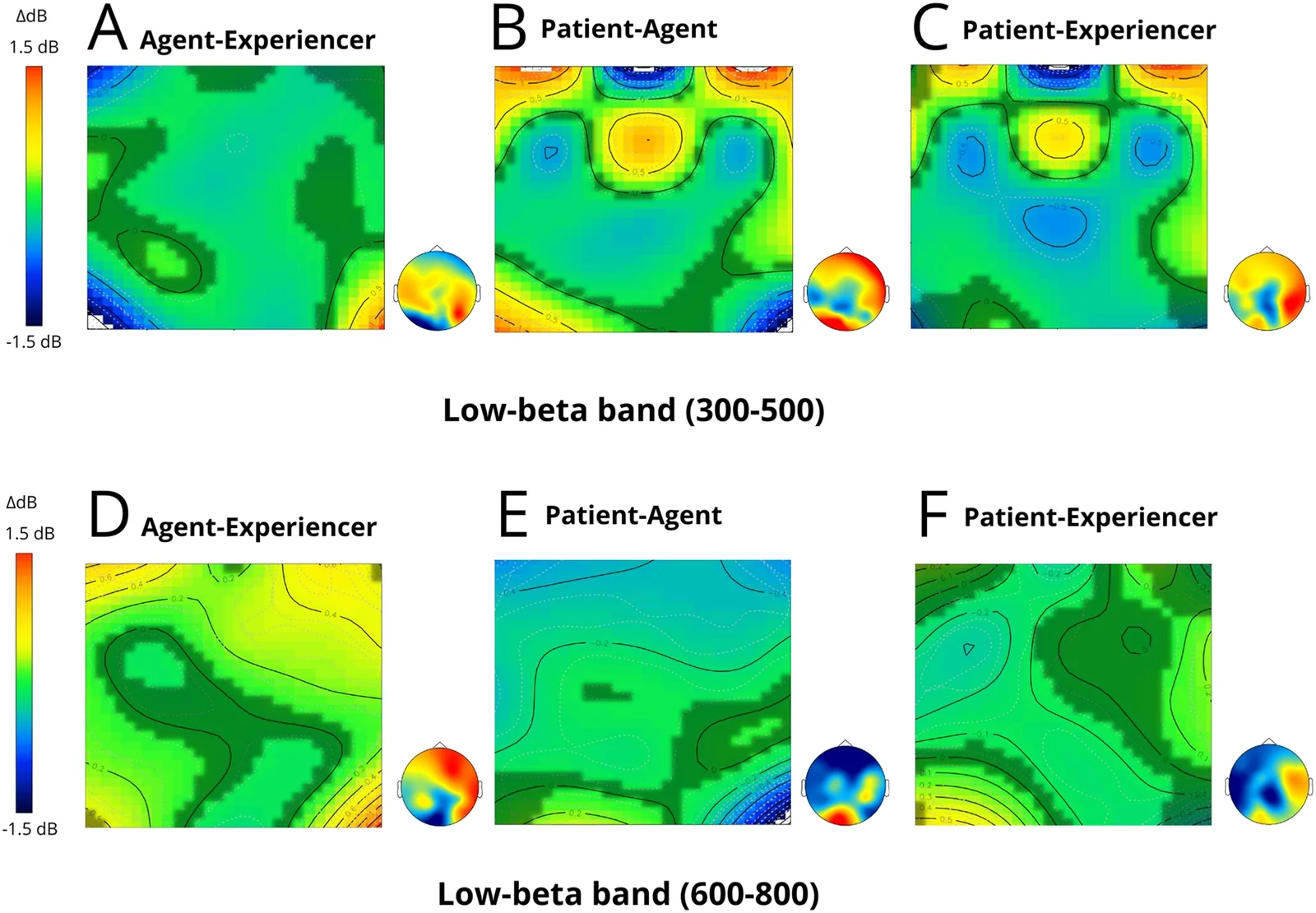
Comparison of power differences in individually defined low-beta band in the 300–500 ms time-window (A, B, C) and in the 600–800 ms time-window (D, E, F) after critical word presentation. Large panels show estimated (GAMM) difference surfaces of the topographic distribution of differences between conditions. Adjacent small scalp plots show the grand-average at the time-frequency point of maximal difference between conditions within each band and time-window. Non-shaded (bright) areas indicate significant differences.

Figure 2 presents the time course of ERPs time-locked to the verb position for nine electrodes electrodes; Figure 3 displays ERP pairwise comparisons of experimental conditions; Figures 4, 5 and 6 show power pairwise comparisons of experimental conditions.

In the 300–500 ms time-window, in ERPs, patients elicited a larger negativity over posterior electrodes compared to agents (Figure 3A, B, C) and experiencers (Figure 3F, G, H). Experiencers elicited a frontal positivity compared to agents (Figure 3K, L, M). In the theta band, patients showed a power increase compared to agents over central electrodes (Figure 4B) and compared to experiencers over central and frontal electrodes (Figure 4C). Agents also revealed a power increase over left-frontal and right-frontal electrodes compared to experiencers (Figure 4A). By contrast, experiencers elicited a power increase over posterior electrodes compared to both agents and patients (Figures 4A, C). Agents also showed this power increase compared to patients but revealing a smaller increase only over right-posterior sites.

In the alpha band, patients elicited a power decrease compared to both agents and experiencers over right posterior electrodes (Figure 5B, C). By contrast, experiencers elicited both power decrease and increase over central and frontal electrodes compared to both agents and patients (Figures 5A, C).

In the low beta band, there was a power decrease for patients compared to agents (Figure 6B) and experiencers (Figure 6C) over frontal and central electrodes, and a power increase over frontal, right, and left-posterior electrodes compared to agents (Figure 6B) and over frontal and right electrodes compared to experiencers (Figure 6C). Experiencers showed a power decrease over right-posterior sites and a power increase over left electrodes compared to agents (Figure 6A).

In the 600–800 ms time-window, results do not indicate a distinct ERP component; rather, they reflect a sustained activity from the N400 time-window. We found that patients elicited a widespread negativity compared to both agents (Figure 3A, D, E) and experiencers (Figure 3F, I, J). In the theta band, patients revealed a highly widespread power increase compared to agents (Figure 4E) and experiencers (Figure 4F). The differences between agents and experiencers in the late time-window followed the pattern reported in the 300–500 time-window but suggested a reduced magnitude of the effects: agents showed a reduced power increase over central electrodes and experiencers a reduced power increase over posterior sites (Figure 4D).

In the alpha band, agents and patients elicited a similar pattern than the one found in the early time-window compared to experiencers: agents and patients elicited a power increase over frontal and left-posterior electrodes, and a power decrease over frontal, central and right-posterior electrodes compared to experiencers (Figures 5D, F). The differences found in the early time-window between agents and patients vanished, revealing only a power decrease of patients over right-frontal electrodes (Figure 5E).

In the low beta band, patients elicited a power decrease over frontal electrodes compared to agents (Figure 6E), and over left-frontal electrodes compared to experiencers (Figure 6F). Experiencers elicited a power decrease over frontal and posterior electrodes compared to agents (Figure 6D).

### Discussion

In the early time-window (300–500 ms), we found that patients elicited a larger negativity compared to both agents and experiencers over posterior electrodes. In previous studies, this N400 component has been associated with a recategorization of the event role category (Bickel et al., 2015; Haupt et al., 2008; Isasi-Isasmendi et al., 2024; Sauppe et al., 2023): since the first argument was transiently interpreted as an agent, when the verb assigns the patient role, the argument needs to be recategorized as a patient. In this work, we replicated the previous N400 effect found between agents and patients, and interestingly, we found the same effect between experiencers and patients. We also found a sustained negativity elicited by patients compared to both agents and experiencers in the 600–800 ms time-window. This negativity was previously reported in event role processing studies (Isasi-Isasmendi et al., 2024; Sauppe et al., 2023), and in other studies of sentence processing (J. M. Schneider & Maguire, 2018; Wang et al., 2012), revealing that N400 effects are not necessarily restricted to the 300–500 ms time-window. This provides evidence that agents and experiencers are processed similarly in the integration of event roles into the syntactic structure. Moreover, this finding aligns with the proto-role approach, which holds that both agents and experiencers are arguments with proto-agent entailments, and they are thus grouped within the proto-agent role category, avoiding the need for reanalysis.

We also found that experiencers elicited a frontal ERP positivity compared to agents in the early time-window. Since experiencers did not elicit the posterior negativity associated with event role recategorization, this frontal positivity may correspond to another processing mechanism, probably due to the lower prototypicality of experiencers compared to agents (Dowty, 1991; Levin & Rappaport Hovav, 2005; Li, 2020; Van Valin, 2004), given that this specific pattern only appears between these two conditions.

Furthermore, in the time-frequency analysis, in the theta band, we found that patients elicited a power increase compared to both agents and experiencers in central electrodes in the early time-window. This increase was stronger and highly widespread in the late time-window. An increase in theta band has been associated with unexpected words and with semantic processing demands (Klimesch, 1999; Mellem et al., 2013; Rommers et al., 2017; Weiss et al., 2000). This result in the theta band can be linked to a reanalysis when encountering a verb selecting a patient role, as the same pattern is found when comparing patients with both agents and experiencers. The processing of verbs assigning a patient role elicited a power increase compared to those selecting agent or experiencer, since these verbs did not match the expectation to assign an argument with proto-agent entailments.

In the alpha band, patients showed a power decrease over right-posterior electrodes in comparison to agents and experiencers, in the early time-window. Additionally, agents and patients elicited a power decrease over more central sites in comparison to experiencers. This effect was more pronounced in the late time-window than in the early time-window. The alpha band has been related to inhibition (Klimesch, 2012; Meyer, 2018), and a power decrease in this band is associated with a release from inhibiting alternative and competing interpretations (Isasi-Isasmendi et al., 2024). According to our results, two types of inhibition could be reflected in the alpha band. On one hand, the elicitation of a power decrease for the patient would be related to the reanalysis previously mentioned. Isasi-Isasmendi et al. (2024) particularly found the same power decrease elicited by ambiguous patients compared to ambiguous agents. They related this result to a release from inhibiting the patient category. On the other hand, the power increase shown by experiencers would align with the inhibition of the prototypical representation of event roles (i.e., agents and patients) (Dowty, 1991; Levin & Rappaport Hovav, 2005; Li, 2020; Van Valin, 2004). Since no previous studies have investigated the power elicitation associated with experiencers, the explanation we offer for this unexpected pattern should be considered tentative.

In the low-beta band, we found that patients elicited a power decrease compared to agents and experiencers over central electrodes, in the early time-window. This pattern did not appear when comparing agents and experiencers. A power decrease in this frequency band has been linked to a change in the current cognitive set to upcoming words that do not match the predicted word, or to semantic violations (Isasi-Isasmendi et al., 2024; Lewis & Bastiaansen, 2015; Meyer, 2018). Moreover, beta oscillations have been related to the N400 component during language processing (Wang et al., 2012). Our findings in this band replicate the same power decrease in the same time-window found in Isasi-Isasmendi et al. (2024), which they associate with a recategorization of the event role category. This power decrease suggests that the processing of patients involves a change in the current active set compared to both agents and experiencers, as it does not fit within the category of proto-agent.

## GENERAL DISCUSSION

In an eye-tracking reading task and in an EEG reading task, we investigated the processing correlates of experiencer-arguments in intransitive sentences in Spanish. We included three conditions: agent, experiencer, and patient subjects. We asked whether experiencers reveal specific processing correlates as agents and patients do, or whether they show similar processing correlates to agents. In the eye-tracking reading experiment, in late eye-tracking measures, we found that agent and experiencer conditions display larger fixation times in the verb and post-verb regions and more regression counts to the verb than the patient condition. In the EEG experiment, results showed that patients elicited a larger N400 component compared to both agents and experiencers. Furthermore, patients elicited a power increase in theta band, and a decrease in alpha and low beta bands compared to both agents and experiencers.

The two methodologies employed allowed us to examine the agent preference from a broad perspective, capturing complementary mechanisms across the two methods. As discussed in the Introduction, the agent preference has been largely investigated with EEG reading experiments, showing a clear result: the processing of patients elicits an N400 component compared to the processing of agents due to the recategorization of the event role. However, to the best of our knowledge, no prior study has investigated it using eye-tracking reading tasks.

In this study, we replicated the N400 effect associated with the agent preference (Bickel et al., 2015; Haupt et al., 2008; Isasi-Isasmendi et al., 2024; Sauppe et al., 2023), as well as the power decrease in alpha and low-beta, also linked with event role recategorization (Isasi-Isasmendi et al., 2024). Importantly, we found evidence that this agent preference extends to experiencer-arguments, since patients elicited an N400 component and a power decrease in alpha and low-beta bands compared to both agents and experiencers.

The patterns of results reported in the eye-tracking experiment fit well with the ones reported in the EEG experiment. In the eye-tracking experiment, we found an attentional preference toward both agents and experiencers compared to patients, reflected in intermediate and late eye-tracking measures. Interestingly, this methodology did not capture the event role recategorization mechanism found in EEG, instead, it reflected attentional preference mechanisms. Previous literature has largely found that agents are attentionally preferred over patients (Cohn & Paczynski, 2013; Gómez-Vidal et al., 2022; Isasi-Isasmendi et al., 2023), even in non-human apes (Brocard et al., 2024, 2025; V. A. D. Wilson et al., 2022, 2024). We observed this attentional preference in Experiment 1, and we found that it extends to experiencers.

Regarding the differences found between agents and experiencers, we also observed that results from both methodologies complement each other. In Experiment 1, in early eye-tracking measures, we observed processing differences between experiencers and both agents and patients. In Experiment 2, we found a similar pattern: an alpha power increase for experiencers compared to both agents and patients. These results are interpreted to reflect a prototypicality effect, showing distinct processing correlates between prototypical roles (i.e., agents and patients) and non-prototypical roles (i.e., experiencers). The fact of finding this effect in both methodologies strengthens the hypothesis that experiencer-arguments are less prototypical roles than agents.

Overall, we found that results from both methodologies complement each other and provide a broad picture of the processing and assignment of event roles. Moreover, our findings open a new line of research examining whether the observed agent preference with experiencer-arguments replicates across languages using these two methodologies.

Regarding event role approaches, our results align with the proto-role approach (Dowty, 1991), since both arguments with proto-agent entailments (i.e., agents and experiencers) are attentionally preferred compared to arguments without proto-agent entailments (i.e., patients). Furthermore, we observed that the proto-agent role is assigned to the first ambiguous noun phrase, involving a recategorization when the argument ends up being a proto-patient. The proto-role approach can also explain the prototypicality effect found based on the Argument Selection Principle that arguments may exhibit distinct degrees of membership into a proto-role. Hence, arguments with only one proto-agent entailment display distinct processing correlates than arguments with all proto-role entailments in early eye-tracking measures and in alpha band.

The proto-role approach (Dowty, 1991) is not the only linguistic approach that can account for these results. For example, the macrorole approach (Foley & Van Valin, 1984; Van Valin, 2004; Van Valin & LaPolla, 1997) also proposes two broad categories of event roles, actor and undergoer. Agents and experiencers are grouped within the actor role and patients under the undergoer category. Hence, this approach can also account for our results related to the attentional preference for both agents and experiencers compared to patients, since both agents and experiencers are considered to be actors. It can also account for the recategorization effect found toward patients, as they are considered undergoers, not actors. Hence, there is an attentional preference toward actors and a preference to assign the actor macrorole to the first ambiguous noun phrase. This approach can also account for the prototypicality effect, since agents and patients are the best exemplifications of actor and undergoer respectively, being experiencers less prototypical actors (Van Valin, 2006; Van Valin & LaPolla, 1997).

However, we still argue that the proto-role approach fits better with evidence that proto-role entailments have psychological reality (Kako, 2006; Rissman & Lupyan, 2022; Vernice & Hartsuiker, 2019). In the macrorole approach, instead, event roles are not decomposed into semantic entailments, rather the event itself is decomposed, with macroroles characterized as “generalizations across classes of specific argument positions in logical structure” (Van Valin & LaPolla, 1997, p. 142).

In the theta system (Reinhart, 2000, 2002, 2016) event roles are also decomposed into semantic features, as in the proto-role approach (Dowty, 1991). But, contrary to the proto-role approach, the theta system proposes an extensive list of event roles, where agents, experiencers, and patients constitute three different role categories. Hence, this approach can account for the processing similarities found between agents and experiencers, since both roles share the [+mental state] semantic feature (i.e., agents–[+cause, +mental state], experiencers–[+mental state]). The theta system can also account for the prototypicality effect: agents are more prototypical arguments for subjecthood than experiencers, because they are characterized by the two semantic features instead of only by one. Nevertheless, this approach cannot account for the recategorization effect, since both agents and experiencers are considered to be two distinct event role categories, and, therefore, a recategorization should be expected when encountering experiencers, as it happens with patients.

Thematic role list approaches (Fillmore, 1968, 1971) differ from the approaches discussed above in the characterization of event roles. In this view, event roles are treated as discrete, non-decomposable categories, with agents and experiencers posited as two distinct role categories. These approaches do not account for the processing similarities we observed between agents and experiencers, because under this view the two roles are treated as distinct, non-decomposable categories, and therefore, they are not predicted to share underlying semantic properties.

Based on our results and on previous psycholinguistic evidence, we consider that event role categories are better understood as broad non-discrete categories decomposed into semantic properties. Hence, although results of Experiments 1 and 2 can also be partially explained by other event role approaches, we consider the proto-role approach to fit better with our results and with previous psychologistic evidence that agents and patients are two core knowledge categories (Rissman & Majid, 2019), and that proto-role entailments have a psychological reality (Kako, 2006; Rissman & Lupyan, 2022; Vernice & Hartsuiker, 2019).

We acknowledge that further research is needed to fully characterize how broad the repertoire of event roles is. To the best of our knowledge, this is the first study to show that agents and experiencers display similar processing correlates, which are different from those displayed by patients. This pattern should be replicated to determine whether the experiencer role category is required in the syntax-semantics interface, or whether only two broad categories are enough. So far, although our prototypicality effect suggests that agents and experiencers are semantically different, our late eye-tracking measures and EEG results reveal that both agents and experiencers display similar processing correlates in the syntax-semantics interface. Importantly, these processing correlates are different from the ones displayed by patients.

All in all, our results provide further evidence of the agent preference hypothesis. During sentence processing, arguments with proto-agent entailments (agents and experiencers) are attentionally preferred compared to arguments without them (patients). Furthermore, ERP findings reveal that the first argument is interpreted as a proto-agent, which involves a recategorization toward a patient when the verb does not select an argument with proto-agent entailments. Interestingly, this recategorization effect does not appear with experiencers. Converging with these results, the time-frequency analysis suggests similarities between agents and experiencers, and differences between them and patients: patients are unexpected, as indicated by a theta power increase; they trigger inhibition of the proto-agent category, as reflected by power increase in alpha band; and they involve a shift in the current cognitive set, evidenced by a power decrease in low beta band.

This evidence is consistent with the proto-role approach (Dowty, 1991). In the syntax-semantics interface, two broad categories of proto-roles are needed, proto-agent and proto-patient. Arguments with proto-agent entailments fit within the proto-agent role; hence, both agents and experiencers are categorized as proto-agents in the interaction with syntax. This evidence broadens the agent preference hypothesis to less prototypical proto-agents, those with the proto-agent entailment of sentience – experiencers.

## CONCLUSION

Our findings from Experiment 1 revealed that agents and experiencers elicited an attentional preference compared to patients. Furthermore, in Experiment 2, we found that patients elicited a recategorization of the event role category due to the agent preference; this recategorization did not appear for experiencers. This suggests that agents and experiencers are grouped under the category of proto-agent as both argument types have proto-agent entailments (Dowty, 1991).

## ACKNOWLEDGMENTS

We thank Itziar Laka for her wholehearted engagement and for the many discussions that helped shape the main ideas of this work. Particularly, her enthusiasm was the spark that set me, Marta, on this fascinating path toward understanding event roles.

## FUNDING INFORMATION

This work was supported by the University of the Basque Country (to Marta Sánchez-López) under Grant PIF21/263; by Grants PID2022-142167NB-I00 (to Marta Sánchez-López) and PID2024-156359NB-I00 (to Mikel Santesteban) funded by MICIU/AEI/10.13039/501100011033 and by ERDF A way of making Europe; and by the Basque Government (to Marta Sánchez-López and Mikel Santesteban) under Grants IT1439-22 and IT1962-26.

## AUTHOR CONTRIBUTIONS

M. S.-L.: Conceptualization; Data curation; Formal analysis; Investigation; Methodology; Resources; Software; Visualization; Writing – Original draft; Writing – Review & editing. A. I.-I.: Conceptualization; Data curation, Formal analysis; Methodology; Visualization; Writing – Review & editing. B. B.: Conceptualization; Formal analysis; Supervision; Writing – Review & editing. M. S.: Conceptualization; Formal analysis; Funding acquisition; Resources; Supervision; Writing – Review & editing.

## DATA AVAILABILITY STATEMENT

The data, analysis scripts, materials, and appendices can be accessed at the following OSF repository: https://osf.io/t9zer/overview?view_only=182b23f8871f4205ba256ac9b13cdc0c.

## Supplementary Material













## References

[bib1] Akaike, H. (1974). A new look at the statistical model identification. IEEE Transactions on Automatic Control, 19(6), 716–723. 10.1109/TAC.1974.1100705

[bib2] Alday, P. M. (2019). How much baseline correction do we need in ERP research? Extended GLM model can replace baseline correction while lifting its limits. Psychophysiology, 56(12), e13451. 10.1111/psyp.13451, 31403187

[bib3] Arel-Bundock, V., Greifer, N., & Heiss, A. (2024). How to interpret statistical models using marginaleffects for R and Python. Journal of Statistical Software, 111(9), 1–32. 10.18637/jss.v111.i09

[bib4] Aurnhammer, C., Delogu, F., Schulz, M., Brouwer, H., & Crocker, M. W. (2021). Retrieval (N400) and integration (P600) in expectation-based comprehension. PLOS ONE, 16(9), e0257430. 10.1371/journal.pone.0257430, 34582472 PMC8478172

[bib5] Bice, K., Yamasaki, B. L., & Prat, C. S. (2020). Bilingual language experience shapes resting-state brain rhythms. Neurobiology of Language, 1(3), 288–318. 10.1162/nol_a_00014, 37215228 PMC10158654

[bib6] Bickel, B., Witzlack-Makarevich, A., Choudhary, K. K., Schlesewsky, M., & Bornkessel-Schlesewsky, I. (2015). The neurophysiology of language processing shapes the evolution of grammar: Evidence from case marking. PLOS ONE, 10(8), e0132819. 10.1371/journal.pone.0132819, 26267884 PMC4534460

[bib7] Bidgood, A., Pine, J. M., Rowland, C. F., & Ambridge, B. (2020). Syntactic representations are both abstract and semantically constrained: Evidence from children’s and adults’ comprehension and production/priming of the English passive. Cognitive Science, 44(9), e12892. 10.1111/cogs.12892, 32918504

[bib8] Bisang, W., Luming, W., & Bornkessel-Schlesewsky, I. (2013). Subjecthood in Chinese: Neurolinguistics meets typology. In Z. Jing-Schmidt (Ed.), Increased empiricism: Recent advances in Chinese linguistics (Vol. 2, pp. 23–48). John Benjamins Publishing Company. 10.1075/scld.2.02bis

[bib9] Bornkessel, I., & Schlesewsky, M. (2006). The role of contrast in the local licensing of scrambling in German: Evidence from online comprehension. Journal of Germanic Linguistics, 18(1), 1–43. 10.1017/S1470542706000018

[bib10] Bornkessel, I., Schlesewsky, M., & Friederici, A. D. (2002). Beyond syntax: Language-related positivities reflect the revision of hierarchies. NeuroReport, 13(3), 361–364. 10.1097/00001756-200203040-00022, 11930138

[bib11] Bornkessel, I., Schlesewsky, M., & Friederici, A. D. (2003). Eliciting thematic reanalysis effects: The role of syntax-independent information during parsing. Language and Cognitive Processes, 18(3), 269–298. 10.1080/01690960244000018

[bib12] Bornkessel-Schlesewsky, I., & Schlesewsky, M. (2009). The role of prominence information in the real-time comprehension of transitive constructions: A cross-linguistic approach. Language and Linguistics Compass, 3(1), 19–58. 10.1111/j.1749-818X.2008.00099.x

[bib13] Bosque, I., Demonte, V., Lázaro Carreter, F., & Pavón Lucero, M. V. (1999). Gramática descriptiva de la lengua española. Espasa.

[bib14] Brennan, J., & Pylkkänen, L. (2010). Processing psych verbs: Behavioural and MEG measures of two different types of semantic complexity. Language and Cognitive Processes, 25(6), 777–807. 10.1080/01690961003616840

[bib15] Brocard, S., Voinov, P. V., Bickel, B., & Zuberbühler, K. (2025). Spontaneous encoding of event roles in hominids. Open Mind: Discoveries in Cognitive Science, 9, 559–575. 10.1162/opmi_a_00202, 40337356 PMC12058332

[bib16] Brocard, S., Wilson, V. A. D., Berton, C., Zuberbühler, K., & Bickel, B. (2024). A universal preference for animate agents in hominids. iScience, 27(6), 109996. 10.1016/j.isci.2024.109996, 38883826 PMC11177197

[bib17] Bürkner, P.-C. (2017). brms: An R package for Bayesian multilevel models using Stan. Journal of Statistical Software, 80(1), 1–28. 10.18637/jss.v080.i01

[bib18] Chaumon, M., Bishop, D. V. M., & Busch, N. A. (2015). A practical guide to the selection of independent components of the electroencephalogram for artifact correction. Journal of Neuroscience Methods, 250, 47–63. 10.1016/j.jneumeth.2015.02.025, 25791012

[bib19] Cohen, M. X. (2014). Analyzing neural time series data: Theory and practice. MIT Press. 10.7551/mitpress/9609.001.0001

[bib20] Cohn, N., & Paczynski, M. (2013). Prediction, events, and the advantage of agents: The processing of semantic roles in visual narrative. Cognitive Psychology, 67(3), 73–97. 10.1016/j.cogpsych.2013.07.002, 23959023 PMC3895484

[bib21] Conklin, K., Pellicer-Sánchez, A., & Carrol, G. (2018). Eye-tracking: A guide for applied linguistics research. Cambridge University Press. 10.1017/9781108233279

[bib22] Corcoran, A. W., Alday, P. M., Schlesewsky, M., & Bornkessel-Schlesewsky, I. (2018). Toward a reliable, automated method of individual alpha frequency (IAF) quantification. Psychophysiology, 55(7), e13064. 10.1111/psyp.13064, 29357113

[bib23] Cuetos, F., Glez-Nosti, M., Barbón, A., & Brysbaert, M. (2011). SUBTLEX-ESP: Spanish word frequencies based on film subtitles. Psicológica, 32(2), 133–143.

[bib24] Cupples, L. (2002). The structural characteristics and on-line comprehension of experiencer-verb sentences. Language and Cognitive Processes, 17(2), 125–162. 10.1080/01690960143000001

[bib25] Delorme, A., & Makeig, S. (2004). EEGLAB: An open source toolbox for analysis of single-trial EEG dynamics including independent component analysis. Journal of Neuroscience Methods, 134(1), 9–21. 10.1016/j.jneumeth.2003.10.009, 15102499

[bib26] Do, M. L., & Kaiser, E. (2022). Sentence formulation is easier when thematic and syntactic prominence align: Evidence from psych verbs. Language, Cognition and Neuroscience, 37(5), 648–670. 10.1080/23273798.2021.2008458

[bib27] Dowty, D. (1991). Thematic proto-roles and argument selection. Language, 67(3), 547–619. 10.1353/lan.1991.0021

[bib28] Erdocia, K., Laka, I., Mestres-Missé, A., & Rodriguez-Fornells, A. (2009). Syntactic complexity and ambiguity resolution in a free word order language: Behavioral and electrophysiological evidences from Basque. Brain and Language, 109(1), 1–17. 10.1016/j.bandl.2008.12.003, 19223065

[bib29] Ferreira, F. (1994). Choice of passive voice is affected by verb type and animacy. Journal of Memory and Language, 33(6), 715–736. 10.1006/jmla.1994.1034

[bib30] Fillmore, C. J. (1968). The case for case. In E. Bach & R. T. Harms (Eds.), Universals in linguistic theory (pp. 1–88). Holt, Rinehart, and Winston.

[bib31] Fillmore, C. J. (1971). Some problems for case grammar. In R. J. O’Brien (Ed.), Report of the 22nd annual round table meeting on linguistics and language studies (pp. 35–56). Georgetown University Press.

[bib32] Foley, W. A., & Van Valin, R. D., Jr. (1984). Functional syntax and universal grammar. Cambridge University Press.

[bib33] Frank, S. L., Otten, L. J., Galli, G., & Vigliocco, G. (2015). The ERP response to the amount of information conveyed by words in sentences. Brain and Language, 140, 1–11. 10.1016/j.bandl.2014.10.006, 25461915

[bib34] Frenzel, S., Schlesewsky, M., & Bornkessel-Schlesewsky, I. (2015). Two routes to actorhood: Lexicalized potency to act and identification of the actor role. Frontiers in Psychology, 6, 1. 10.3389/fpsyg.2015.00001, 25688217 PMC4311632

[bib35] Galazka, M., & Nyström, P. (2016). Infants’ preference for individual agents within chasing interactions. Journal of Experimental Child Psychology, 147, 53–70. 10.1016/j.jecp.2016.02.010, 27017143

[bib36] Gattei, C. A., Dickey, M. W., Wainselboim, A. J., & París, L. (2015). The thematic hierarchy in sentence comprehension: A study on the interaction between verb class and word order in Spanish. Quarterly Journal of Experimental Psychology, 68(10), 1981–2007. 10.1080/17470218.2014.1000345, 25529525

[bib37] Gattei, C. A., Sevilla, Y., Tabullo, Á. J., Wainselboim, A. J., París, L. A., & Shalom, D. E. (2018). Prominence in Spanish sentence comprehension: An eye-tracking study. Language, Cognition and Neuroscience, 33(5), 587–607. 10.1080/23273798.2017.1397278

[bib38] Gattei, C. A., Tabullo, Á., París, L., & Wainselboim, A. J. (2015). The role of prominence in Spanish sentence comprehension: An ERP study. Brain and Language, 150, 22–35. 10.1016/j.bandl.2015.08.001, 26291770

[bib39] Gennari, S., & MacDonald, M. C. (2009). Linking production and comprehension processes: The case of relative clauses. Cognition, 111(1), 1–23. 10.1016/j.cognition.2008.12.006, 19215912

[bib40] Gennari, S., & Poeppel, D. (2003). Processing correlates of lexical semantic complexity. Cognition, 89(1), B27–B41. 10.1016/S0010-0277(03)00069-6, 12893127

[bib41] Gómez-Vidal, B. (2024). Processing argument structure: Eye-tracking evidence from Spanish native speakers in the Visual World Paradigm [Unpublished doctoral dissertation]. University of the Basque Country.

[bib42] Gómez-Vidal, B., Arantzeta, M., Laka, J. P., & Laka, I. (2022). Subjects are not all alike: Eye-tracking the agent preference in Spanish. PLOS ONE, 17(8), e0272211. 10.1371/journal.pone.0272211, 35921377 PMC9348668

[bib43] Haupt, F. S., Schlesewsky, M., Roehm, D., Friederici, A. D., & Bornkessel-Schlesewsky, I. (2008). The status of subject–object reanalyses in the language comprehension architecture. Journal of Memory and Language, 59(1), 54–96. 10.1016/j.jml.2008.02.003

[bib44] Huang, Y. T., Zheng, X., Meng, X., & Snedeker, J. (2013). Children’s assignment of grammatical roles in the online processing of Mandarin passive sentences. Journal of Memory and Language, 69(4), 589–606. 10.1016/j.jml.2013.08.002, 24376303 PMC3872120

[bib45] Huber, E., Sauppe, S., Isasi-Isasmendi, A., Bornkessel-Schlesewsky, I., Merlo, P., & Bickel, B. (2024). Surprisal from language models can predict ERPs in processing predicate-argument structures only if enriched by an agent preference principle. Neurobiology of Language, 5(1), 167–200. 10.1162/nol_a_00121, 38645615 PMC11025647

[bib46] Isasi-Isasmendi, A., Andrews, C., Flecken, M., Laka, I., Daum, M. M., Meyer, M., Bickel, B., & Sauppe, S. (2023). The agent preference in visual event apprehension. Open Mind: Discoveries in Cognitive Science, 7, 240–282. 10.1162/opmi_a_00083, 37416075 PMC10320828

[bib47] Isasi-Isasmendi, A., Sauppe, S., Andrews, C., Laka, I., Meyer, M., & Bickel, B. (2024). Incremental sentence processing is guided by a preference for agents: EEG evidence from Basque. Language, Cognition and Neuroscience, 39(1), 76–97. 10.1080/23273798.2023.2250023

[bib48] Jackendoff, R. S. (1990). Semantic structures. MIT Press.

[bib49] Kako, E. (2006). Thematic role properties of subjects and objects. Cognition, 101(1), 1–42. 10.1016/j.cognition.2005.08.002, 16289069

[bib50] Katsika, A., Braze, D., Deo, A., & Piñango, M. M. (2012). Complement coercion: Distinguishing between type-shifting and pragmatic inferencing. Mental Lexicon, 7(1), 58–76. 10.1075/ml.7.1.03kat, 26925175 PMC4767168

[bib51] Klimesch, W. (1999). EEG alpha and theta oscillations reflect cognitive and memory performance: A review and analysis. Brain Research Reviews, 29(2–3), 169–195. 10.1016/S0165-0173(98)00056-3, 10209231

[bib52] Klimesch, W. (2012). α-band oscillations, attention, and controlled access to stored information. Trends in Cognitive Sciences, 16(12), 606–617. 10.1016/j.tics.2012.10.007, 23141428 PMC3507158

[bib53] Kruschke, J. K. (2015). Doing Bayesian data analysis: A tutorial introduction with R, JAGS, and Stan (2nd ed.). Elsevier/Academic Press.

[bib54] Laka, I., & Erdozia, K. (2012). Linearization preferences given “free word order”; subject preferences given ergativity: A look at Basque. In E. Torrego (Ed.), Of grammar, words, and verses: In honor of Carlos Piera (Vol. 8, pp. 115–142). John Benjamins Publishing Company. 10.1075/lfab.8.09lak

[bib55] Lamers, M. J. A. (2012). Argument linearization in Dutch: A multi-factorial approach. In M. Lamers & P. de Swart (Eds.), Case, word order and prominence: Interacting cues in language production and comprehension (Vol. 40, pp. 121–144). Springer. 10.1007/978-94-007-1463-2_6

[bib56] Levin, B. (1993). English verb classes and alternations: A preliminary investigation. University of Chicago Press.

[bib57] Levin, B., & Rappaport Hovav, M. (2005). Argument realization. Cambridge University Press. 10.1017/CBO9780511610479

[bib58] Lewis, A. G., & Bastiaansen, M. (2015). A predictive coding framework for rapid neural dynamics during sentence-level language comprehension. Cortex, 68, 155–168. 10.1016/j.cortex.2015.02.014, 25840879

[bib59] Li, C. (2020). Causer and causee as two higher-ranked thematic roles. Language and Linguistics, 21(4), 601–635. 10.1075/lali.00072.li

[bib60] Ma, Y., Buccola, B., Wang, Z., Cousins, S., Godfroid, A., & Beretta, A. (2023). Expressions with aspectual verbs elicit slower reading times than those with psychological verbs: An eye-tracking study in Mandarin Chinese. Journal of Psycholinguistic Research, 52(1), 179–215. 10.1007/s10936-022-09846-y, 35230571

[bib61] Manouilidou, C., & de Almeida, R. G. (2013). Processing correlates of verb typologies: Investigating internal structure and argument realization. Linguistics, 51(4), 767–792. 10.1515/ling-2013-0026

[bib62] Manouilidou, C., de Almeida, R. G., Schwartz, G., & Nair, N. P. V. (2009). Thematic roles in Alzheimer’s disease: Hierarchy violations in psychological predicates. Journal of Neurolinguistics, 22(2), 167–186. 10.1016/j.jneuroling.2008.10.002

[bib63] Martinez de la Hidalga, G., Zawiszewski, A., & Laka, I. (2019). *Eppur non si muove*: Experimental evidence for the Unaccusative Hypothesis and distinct ϕ-feature processing in Basque. Glossa, 4(1), 120. 10.5334/gjgl.829

[bib64] McElreath, R. (2020). Statistical rethinking: A Bayesian course with examples in R and Stan (2nd ed.). Chapman and Hall/CRC. 10.1201/9780429029608

[bib65] Mellem, M. S., Friedman, R. B., & Medvedev, A. V. (2013). Gamma- and theta-band synchronization during semantic priming reflect local and long-range lexical–semantic networks. Brain and Language, 127(3), 440–451. 10.1016/j.bandl.2013.09.003, 24135132 PMC3864756

[bib66] Meyer, L. (2018). The neural oscillations of speech processing and language comprehension: State of the art and emerging mechanisms. European Journal of Neuroscience, 48(7), 2609–2621. 10.1111/ejn.13748, 29055058

[bib67] Michaelov, J. A., Bardolph, M. D., Van Petten, C. K., Bergen, B. K., & Coulson, S. (2024). Strong prediction: Language model surprisal explains multiple N400 effects. Neurobiology of Language, 5(1), 107–135. 10.1162/nol_a_00105, 38645623 PMC11025652

[bib68] Newmeyer, F. J. (2010). On comparative concepts and descriptive categories: A reply to Haspelmath. Language, 86(3), 688–695. 10.1353/lan.2010.0000

[bib69] Nicenboim, B., Schad, D. J., & Vasishth, S. (2025). Introduction to Bayesian data analysis for cognitive science (1st ed.). Chapman and Hall/CRC. 10.1201/9780429342646

[bib70] Nolan, H., Whelan, R., & Reilly, R. B. (2010). *FASTER*: Fully automated statistical thresholding for EEG artifact rejection. Journal of Neuroscience Methods, 192(1), 152–162. 10.1016/j.jneumeth.2010.07.015, 20654646

[bib71] Oostenveld, R., Fries, P., Maris, E., & Schoffelen, J.-M. (2011). FieldTrip: Open source software for advanced analysis of MEG, EEG, and invasive electrophysiological data. Computational Intelligence and Neuroscience, 2011, 156869. 10.1155/2011/156869, 21253357 PMC3021840

[bib72] R Core Team. (2025). R: A language and environment for statistical computing (Version 4.1.1) [Computer software]. R Foundation for Statistical Computing. https://www.R-project.org/

[bib73] Ramchand, G. C. (2008). Verb meaning and the lexicon: A first phase syntax (1st ed.). Cambridge University Press. 10.1017/CBO9780511486319

[bib74] Reinhart, T. (2000). The theta system: Syntactic realization of verbal concepts (OTS Working Papers in Linguistics). Utrecht Institute of Linguistics, Utrecht University.

[bib75] Reinhart, T. (2002). The theta system—An overview. Theoretical Linguistics, 28(3), 229–290. 10.1515/thli.28.3.229

[bib76] Reinhart, T. (2016). The theta system: Syntactic realization of verbal concepts. In M. Everaert, M. Marelj, & E. Reuland (Eds.), Concepts, syntax, and their interface: The theta system (pp. 1–158). MIT Press. 10.7551/mitpress/8496.003.0003

[bib78] Rissman, L., & Lupyan, G. (2022). A dissociation between conceptual prominence and explicit category learning: Evidence from agent and patient event roles. Journal of Experimental Psychology: General, 151(7), 1707–1732. 10.1037/xge0001146, 34766824

[bib79] Rissman, L., & Majid, A. (2019). Thematic roles: Core knowledge or linguistic construct? Psychonomic Bulletin & Review, 26(6), 1850–1869. 10.3758/s13423-019-01634-5, 31290008 PMC6863944

[bib80] Rommers, J., Dickson, D. S., Norton, J. J. S., Wlotko, E. W., & Federmeier, K. D. (2017). Alpha and theta band dynamics related to sentential constraint and word expectancy. Language, Cognition and Neuroscience, 32(5), 576–589. 10.1080/23273798.2016.1183799, 28761896 PMC5533299

[bib81] Rozwadowska, B. (1988). Thematic restrictions on derived nominals. In W. Wilkins (Ed.), Thematic relations (pp. 147–165). Brill. 10.1163/9789004373211_010

[bib82] Rozwadowska, B. (1989). Are thematic relations discrete? In R. Corrigan, F. Eckman, & M. Noonan (Eds.), Linguistic categorization (pp. 115–130). John Benjamins Publishing. 10.1075/cilt.61.09roz

[bib83] Sánchez-López, M. (2026). Processing proto-agents and proto-patients: Eye-tracking and EEG evidence in Spanish [Doctoral dissertation]. University of the Basque Country. https://osf.io/wnar8/overview?view_only=fda96c2cf9244181baffce94f1c03c54

[bib84] Sauppe, S., Næss, Å., Roversi, G., Meyer, M., Bornkessel-Schlesewsky, I., & Bickel, B. (2023). An agent-first preference in a patient-first language during sentence comprehension. Cognitive Science, 47(9), e13340. 10.1111/cogs.13340, 37715510

[bib85] Schneider, J. M., & Maguire, M. J. (2018). Identifying the relationship between oscillatory dynamics and event-related responses. International Journal of Psychophysiology, 133, 182–192. 10.1016/j.ijpsycho.2018.07.002, 29981766

[bib86] Schneider, W., Eschman, A., & Zuccolotto, A. (2012). E-prime reference guide [Computer software]. Psychology Software Tools, Inc.

[bib87] Schotter, E. R., & Dillon, B. (2025). A beginner’s guide to eye tracking for psycholinguistic studies of reading. Behavior Research Methods, 57(2), 68. 10.3758/s13428-024-02572-4, 39843882

[bib88] SR Research. (2020). FAQ: How do I extract reading measures from data viewer? SR Support Forum. https://sr-research.com/support/thread-336.html

[bib89] SR Research Ltd. (2022). DataViewer (Version 4.2.1) [Computer software]. SR Research Ltd. https://www.sr-research.com/data-viewer/

[bib90] Thompson, C. K., & Lee, M. (2009). Psych verb production and comprehension in agrammatic Broca’s aphasia. Journal of Neurolinguistics, 22(4), 354–369. 10.1016/j.jneuroling.2008.11.003, 20174592 PMC2824436

[bib77] van Rij, J., Wieling, M., Baayen, R. H., & van Rijn, H. (2020). itsadug: Interpreting time series and autocorrelated data using GAMMs [Computer software]. https://cran.r-project.org/package=itsadug

[bib91] Van Valin, R. D., Jr. (1999). Generalized semantic roles and the syntax-semantics interface. In F. Corblin, C. Dobrovie-Sorin, & J.-M. Marandin (Eds.), Empirical issues in formal syntax and semantics 2 (pp. 373–389). Thesus.

[bib92] Van Valin, R. D., Jr. (2004). Semantic macroroles in role and reference grammar. In R. Kailuweit & M. Hummel (Eds.), Semantische rollen (pp. 62–82). Gunter Narr Verlag.

[bib93] Van Valin, R. D., Jr. (2006). Semantic macroroles and language processing. In I. Bornkessel, M. Schlesewsky, B. Comrie, & A. D. Friederici (Eds.), Semantic role universals and argument linking: Theoretical, typological, and psycholinguistic perspectives (pp. 263–302). Mouton de Gruyter. 10.1515/9783110219272.263

[bib94] Van Valin, R. D., Jr., & LaPolla, R. J. (1997). Syntax: Structure, meaning, and function. Cambridge University Press. 10.1017/CBO9781139166799

[bib95] Vehtari, A., Gelman, A., & Gabry, J. (2017). Practical Bayesian model evaluation using leave-one-out cross-validation and WAIC. Statistics and Computing, 27(5), 1413–1432. 10.1007/s11222-016-9696-4

[bib96] Vela-Plo, L., Erdocia, K., & Laka, I. (2022). Agents strongly preferred: Ambiguity processing in Basque. In K. Bilbao, A. Odria, A. Berro, J. Landa, & B. Fernández (Eds.), Papers on Basque linguistics in honour of Jon Franco (pp. 167–194). Deusto University Press.

[bib97] Vernice, M., & Hartsuiker, R. J. (2019). Mapping thematic roles onto grammatical functions in sentence production: Evidence from structural priming in Italian. Journal of Cultural Cognitive Science, 3(S1), 39–64. 10.1007/s41809-019-00044-2

[bib98] Wang, L., Jensen, O., van den Brink, D., Weder, N., Schoffelen, J.-M., Magyari, L., Hagoort, P., & Bastiaansen, M. (2012). Beta oscillations relate to the N400m during language comprehension. Human Brain Mapping, 33(12), 2898–2912. 10.1002/hbm.21410, 22488914 PMC6870343

[bib99] Warren, T., White, S. J., & Reichle, E. D. (2009). Investigating the causes of wrap-up effects: Evidence from eye movements and E–Z Reader. Cognition, 111(1), 132–137. 10.1016/j.cognition.2008.12.011, 19215911 PMC2682724

[bib100] Weiss, S., Müller, H. M., & Rappelsberger, P. (2000). Theta synchronization predicts efficient memory encoding of concrete and abstract nouns. NeuroReport, 11(11), 2357–2361. 10.1097/00001756-200008030-00005, 10943685

[bib101] Wickham, H., Chang, W., Henry, L., Pedersen, T. L., Takahashi, K., Wilke, C., Woo, K., Yutani, H., Dunnington, D., & van den Brand, T. (2007). ggplot2: Create elegant data visualisations using the grammar of graphics (Version 3.5.2) [Dataset]. 10.32614/CRAN.package.ggplot2

[bib102] Wilson, M., & Dillon, B. (2022). Alignment between thematic roles and grammatical functions facilitates sentence processing: Evidence from experiencer verbs. SSRN Electronic Journal. 10.2139/ssrn.4235952

[bib103] Wilson, V. A. D., Sauppe, S., Brocard, S., Ringen, E., Daum, M. M., Wermelinger, S., Gu, N., Andrews, C., Isasi-Isasmendi, A., Bickel, B., & Zuberbühler, K. (2024). Humans and great apes visually track event roles in similar ways. PLOS Biology, 22(11), e3002857. 10.1371/journal.pbio.3002857, 39591401 PMC11593759

[bib104] Wilson, V. A. D., Zuberbühler, K., & Bickel, B. (2022). The evolutionary origins of syntax: Event cognition in nonhuman primates. Science Advances, 8(25), eabn8464. 10.1126/sciadv.abn8464, 35731868 PMC9216513

[bib105] Wood, S. N. (2004). Stable and efficient multiple smoothing parameter estimation for generalized additive models. Journal of the American Statistical Association, 99(467), 673–686. 10.1198/016214504000000980

[bib106] Wood, S. N. (2011). Fast stable restricted maximum likelihood and marginal likelihood estimation of semiparametric generalized linear models. Journal of the Royal Statistical Society, Series B: Statistical Methodology, 73(1), 3–36. 10.1111/j.1467-9868.2010.00749.x

[bib107] Yao, Y., Vehtari, A., Simpson, D., & Gelman, A. (2018). Using stacking to average Bayesian predictive distributions (with discussion). Bayesian Analysis, 13(3), 917–1007. 10.1214/17-BA1091

[bib108] Zehr, J., & Schwarz, F. (2022). PennController for internet based experiments (IBEX) [Computer software]. Open Science Framework. 10.17605/OSF.IO/MD832

[bib109] Zeyrek, D., & Acartürk, C. (2014). The distinction between unaccusative and unergative verbs in Turkish: An offline and an eye tracking study of split intransitivity. In P. Bello, M. Guarini, M. McShane, & B. Scassellati (Eds.), Proceedings of the 36th Annual Conference of the Cognitive Science Society (pp. 1832–1837). Cognitive Science Society.

