# Supplementary material for: Broadening the Agent Preference Hypothesis Through Experiencers: Eye-Tracking and EEG Evidence of Proto-Agents and Proto-Patients
Source: Open Mind (Camb). 2026 Jul 17;10:1092–117. doi: 10.1162/OPMI.a.372 (PMC13518077; doi:10.1162/OPMI.a.372)
Supplement: Supplementary file 1 [file opmi-10-1092-s001.pdf]

Pairwise comparisons of length and frequency of verbs used in Experiments 1 and 2

Δ	Length<br>Estimate [CrI]	Frequency<br>Estimate [CrI]
Agent – experiencer	0.22 [-0.85, 1.25]	0.04 [-0.23, 0.33]
Agent – patient	0.03 [-1.18, 1.25]	-0.03 [-0.22, 0.31]
Experiencer – patient	-0.15 [-1.38, 1.05]	-0.01 [-0.31, 0.28]
