# Supplementary material for: Broadening the Agent Preference Hypothesis Through Experiencers: Eye-Tracking and EEG Evidence of Proto-Agents and Proto-Patients
Source: Open Mind (Camb). 2026 Jul 17;10:1092–117. doi: 10.1162/OPMI.a.372 (PMC13518077; doi:10.1162/OPMI.a.372)
Supplement: Supplementary file 2 [file opmi-10-1092-s002.pdf]

Model comparison of Experiment 1

The stacking weights derived from loo-cv for each eye-tracking measure and region. Highest-weighted models are in bold.

Measure	Null model	Model surprisal	Model predictor	Full model
**Gaze Duration**				
Subject region	**0.940**	0.060	0.000	0.000
Pre-verb region	0.137	0.146	**0.717**	0.000
Verb region	0.060	0.000	0.000	**0.940**
Post-verb region	0.221	**0.608**	0.171	0.000
**Regression Path Duration**				
Subject region	**0.708**	0.287	0.005	0.000
Pre-verb region	0.429	**0.571**	0.000	0.000
Verb region	0.019	0.047	0.102	**0.833**
Post-verb region	0.000	0.411	**0.583**	0.007
**Re-Reading Duration**				
Pre-verb region	**0.698**	0.302	0.000	0.000
Verb region	0.000	0.362	**0.634**	0.004
Post-verb region	0.114	**0.786**	0.000	0.100
**Regression Out Count**				
Pre-verb region	**1.000**	0.000	0.000	0.000
Verb region	**0.926**	0.000	0.074	0.000
Post-verb region	0.299	0.000	**0.701**	0.000
**Regression In Count**				
Subject region	**0.566**	0.434	0.000	0.000
Pre-verb region	0.199	**0.665**	0.117	0.020
Verb region	0.350	0.001	**0.650**	0.000
Post-verb region	0.000	**1.000**	0.000	0.000
**Total Duration**				
Subject region	**0.680**	0.320	0.000	0.000
Pre-verb region	**0.598**	0.393	0.000	0.009
Verb region	0.000	0.031	0.155	**0.814**
Post-verb region	0.000	**0.601**	0.271	0.127
