# Supplementary material for: Broadening the Agent Preference Hypothesis Through Experiencers: Eye-Tracking and EEG Evidence of Proto-Agents and Proto-Patients
Source: Open Mind (Camb). 2026 Jul 17;10:1092–117. doi: 10.1162/OPMI.a.372 (PMC13518077; doi:10.1162/OPMI.a.372)
Supplement: Supplementary file 3 [file opmi-10-1092-s003.pdf]

ERP time course analysis

In addition to the main ERP analyses restricted to two predefined time windows, we conducted an additional time course analysis spanning the interval from –100 to 896 ms relative to the onset of the critical verb. This analysis focused on a central–posterior region of interest, including electrodes Cz, CP1, CP2, C3, C4, Pz, P4, P8, and CP6. The model that included experimental condition as a predictor provided a significantly better fit than the null model ($F(2.84) = 1360.6$, $p < .001$).

Pairwise comparisons between conditions (agent vs. experiencer, agent vs. patient, and experiencer vs. patient) revealed consistent differences between 300-500 ms and 600-800 ms following the verb onset. This extended time course analysis supports the selection of time windows used in the main analyses (centered on the N400 and P600 components). Some earlier effects were also detected though the interpretation of very early ERP responses remains controversial (Nieuwland, 2019, Isasi-Isasmendi et al., 2024). It is more plausible that such early differences arise from visual or lexical characteristics of the verb stimuli, since the verb sets differ across conditions.

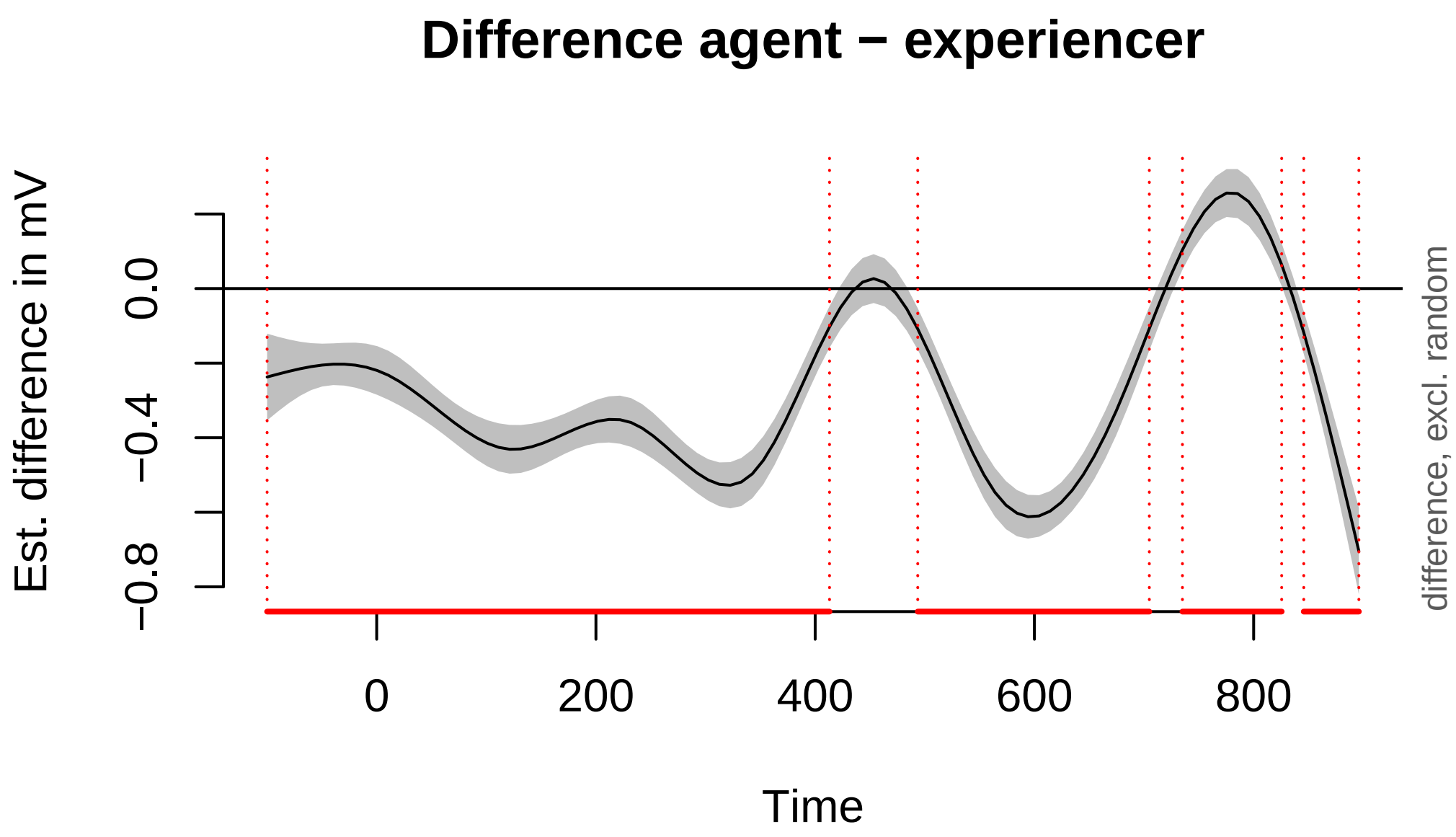
Difference agent − experiencer
Est. difference in mV
0.0
−0.4
−0.8
0
200
400
600
800
Time
difference, excl. random

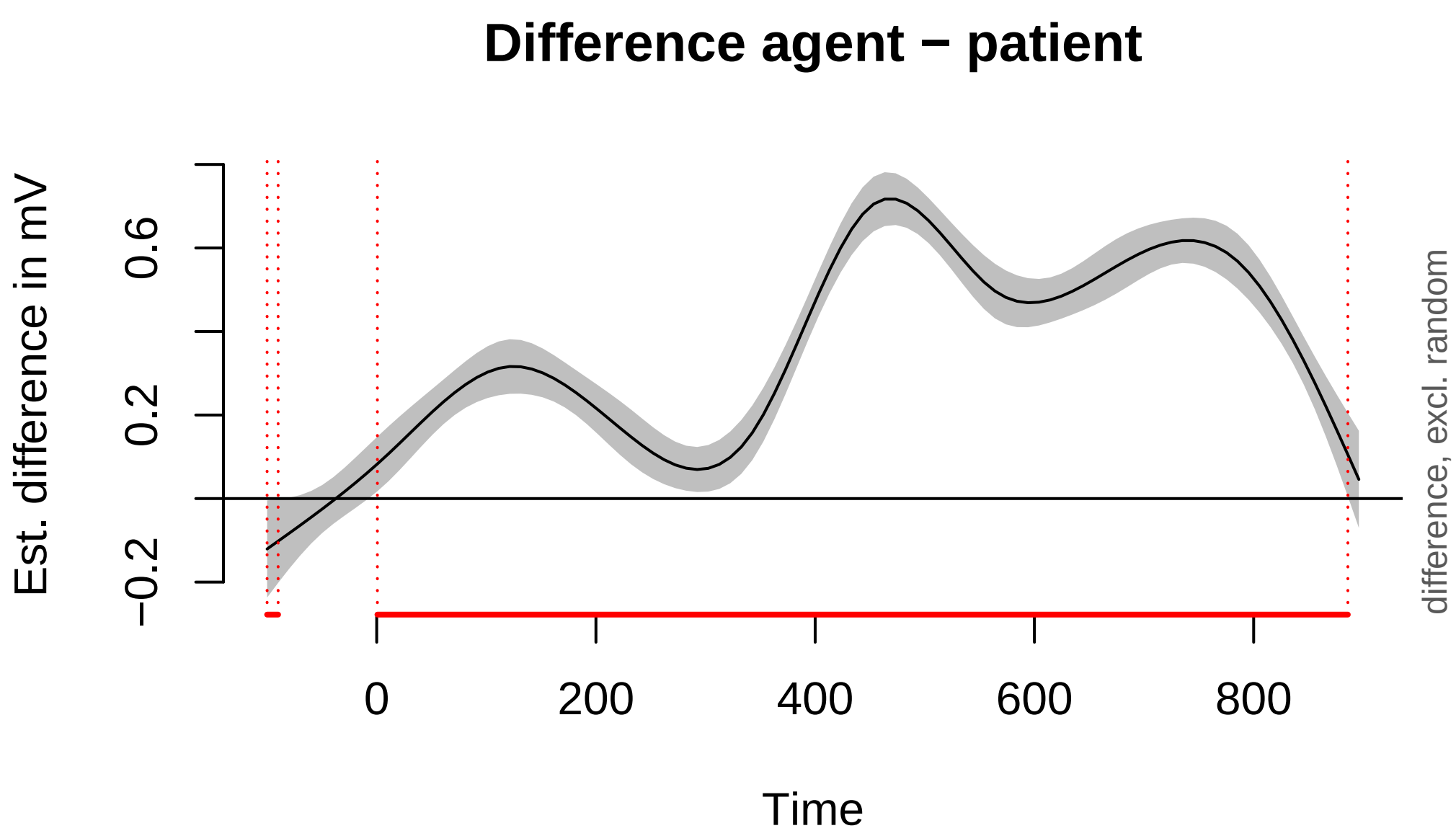
Difference agent − patient
Est. difference in mV
0.6
0.2
−0.2
0
200
400
600
800
Time
difference, excl. random

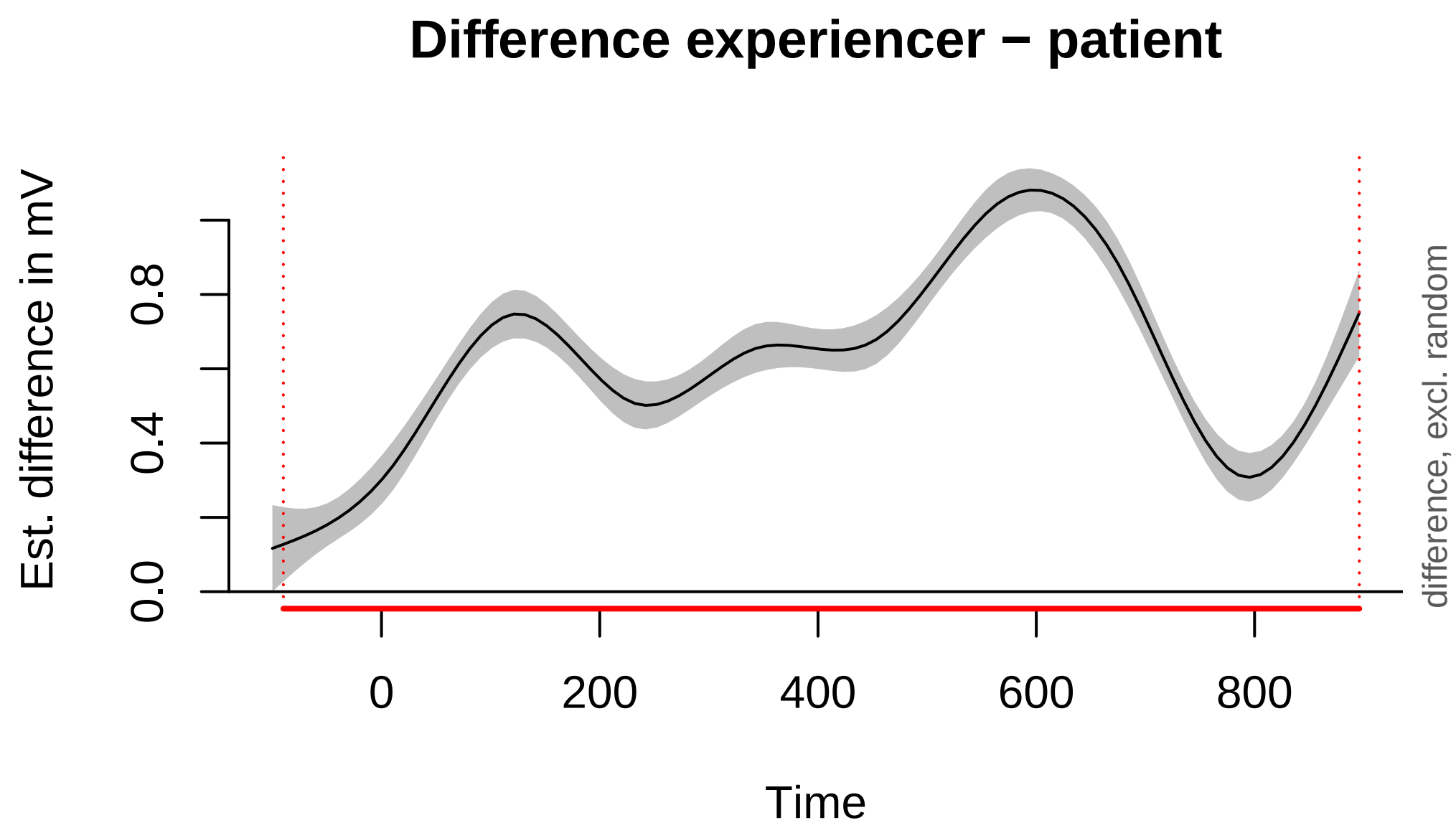

Figure 1. Temporal distribution of amplitude differences between conditions, extracted from the GAMM model. Red areas indicate where the difference between conditions is significant (confidence interval does not include zero).
