# Supplementary material for: Broadening the Agent Preference Hypothesis Through Experiencers: Eye-Tracking and EEG Evidence of Proto-Agents and Proto-Patients
Source: Open Mind (Camb). 2026 Jul 17;10:1092–117. doi: 10.1162/OPMI.a.372 (PMC13518077; doi:10.1162/OPMI.a.372)
Supplement: Supplementary file 4 [file opmi-10-1092-s004.pdf]

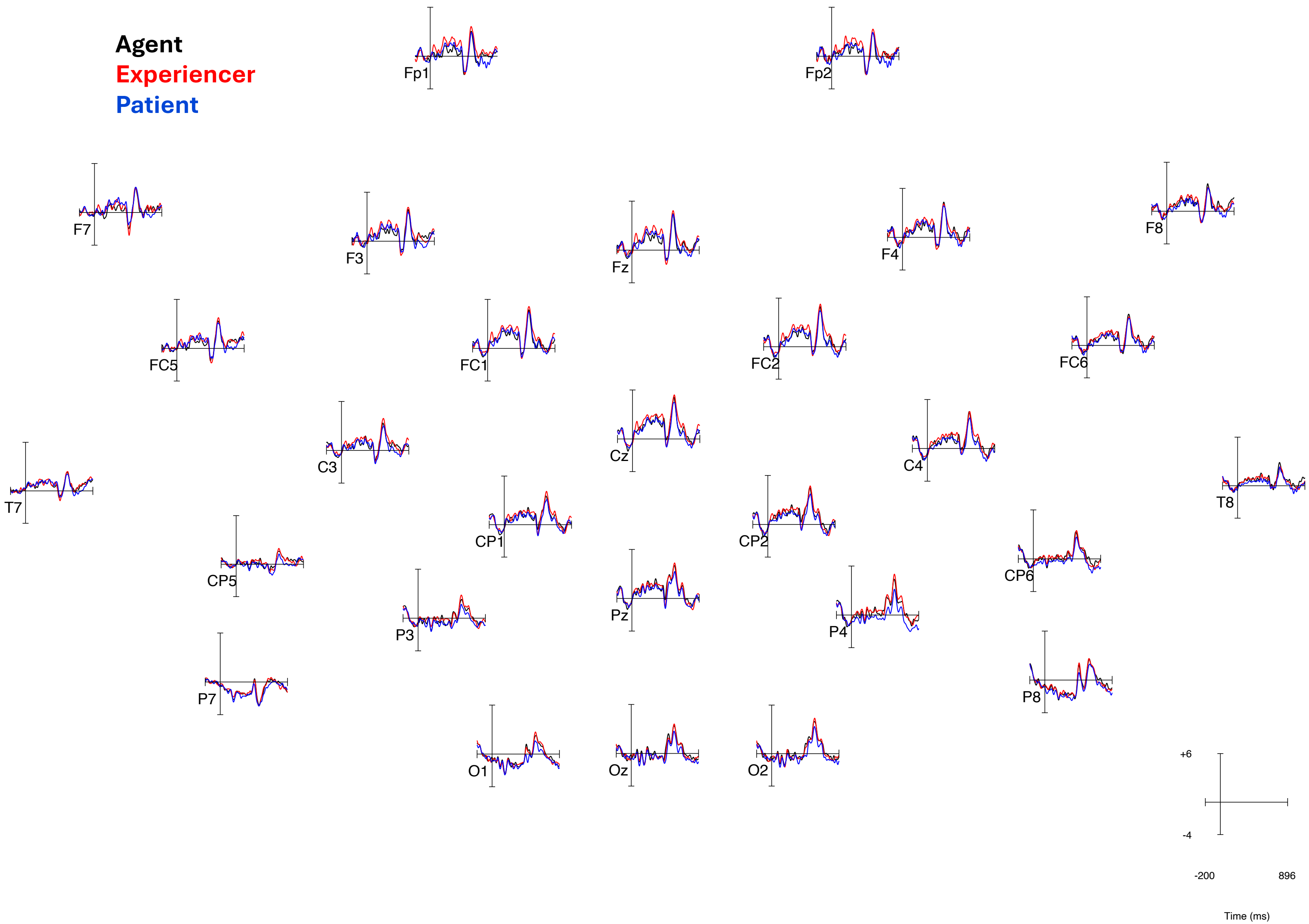
Agent
Experiencer
Patient
Fp1
Fp2
F7
F3
Fz
F4
F8
FC5
FC1
FC2
FC6
T7
C3
Cz
C4
T8
CP5
CP1
CP2
CP6
P7
P3
Pz
P4
P8
O1
Oz
O2
+6
-4
-200
896
Time (ms)
