# Supplementary material for: Broadening the Agent Preference Hypothesis Through Experiencers: Eye-Tracking and EEG Evidence of Proto-Agents and Proto-Patients
Source: Open Mind (Camb). 2026 Jul 17;10:1092–117. doi: 10.1162/OPMI.a.372 (PMC13518077; doi:10.1162/OPMI.a.372)
Supplement: Supplementary file 5 [file opmi-10-1092-s005.pdf]

Eye-tracking measures

The Gaze Duration variable, an early measure, captures the time spent fixating the target region during the first pass, before looking at any region to the right or to the left. Re-reading Duration, and Regression Path Duration are intermediate measures. The Regression Path Duration variable reflects the time participants have fixated on a target region before having fixated any region on the right, but it may include regressions from the left. The Re-reading Duration is calculated as the difference between Regression Path Duration and Gaze Duration. The Regression Out Count variable measures the number of regressions from the target region to a region on the left before any rightward movement while the Regression In Count variable tracks entries into the target region from the right. The Total Duration variable, a late measure, includes the total time participants have been looking at the target region, including re-reading times from both sides. (Conklin et al., 2018; SR Research, 2020). See Figure 1 and Table 1 for an exemplification of an eye-movement pattern during reading and their corresponding eye-tracking variables.

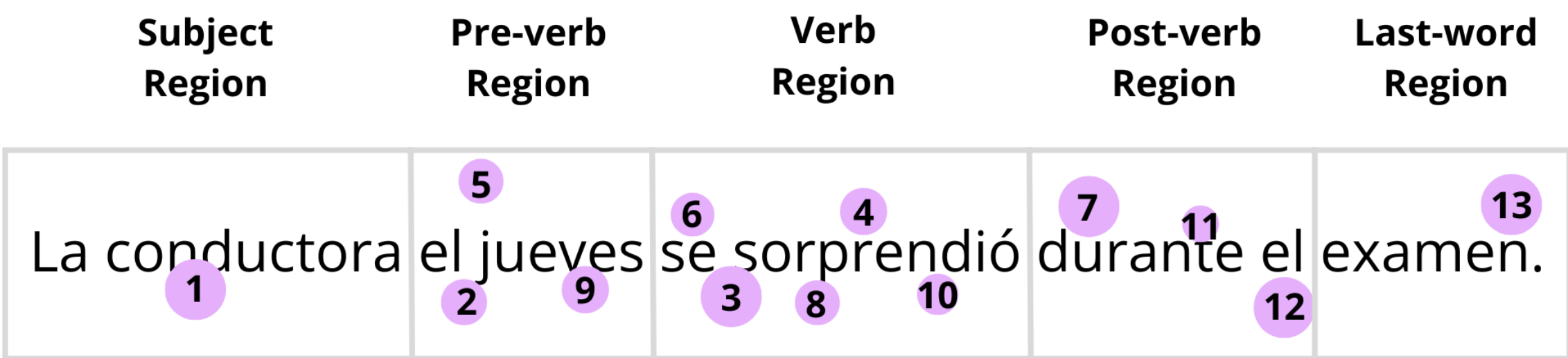

Figure 1: This figure illustrates a made-up example of eye-movement pattern during reading an experimental sentence. Each dot represents a fixation, the bigger it is, the more milliseconds a participant has spent on this fixation. The number in the dots indicates the order of the fixations during sentence reading.

Table 1: The number of corresponding fixations of each eye-tracking measure with the fixation numbers in the verb region of Figure 1. In all duration measures (i.e., Gaze Duration, Regression Path Duration, Re-reading Duration, and Total Duration), the sum of the fixations results in a number in milliseconds (e.g., 350 ms). In the count measures (i.e., Regression Out Count and Regression In Count), the sum of fixations results in a specific number of counts (e.g., 2 counts for Regression Out Count).

**Eye-tracking measures**	**Fixation numbers**
Gaze Duration	3 + 4
Regression Path Duration	3 + 4 + 6
Re-reading Duration	6
Regression Out Count	4 + 8
Regression In Count	8
Total Duration	3 + 4 + 6 + 8 + 10
