# Supplementary material for: Broadening the Agent Preference Hypothesis Through Experiencers: Eye-Tracking and EEG Evidence of Proto-Agents and Proto-Patients
Source: Open Mind (Camb). 2026 Jul 17;10:1092–117. doi: 10.1162/OPMI.a.372 (PMC13518077; doi:10.1162/OPMI.a.372)
Supplement: Supplementary file 6 [file opmi-10-1092-s006.pdf]

Model comparison of Experiment 2

Stacking weights derived from loo-cv for ERPs. Highest-weighted models are in bold.

ERP models

Time-window	Null model	Model surprisal	Model predictor	Full model
300-500 ms	0.020	0.000	**0.975**	0.005
600-800 ms	0.001	0.300	**0.676**	0.023

For TFA models, the AIC values are reported here, with lower values (shown in bold) indicating better model fit.

TFA models

Theta band

Time-window	Null model	Model surprisal	Model predictor	Full model
300-500 ms	7985389	7985388	**7984991**	7984994
600-800 ms	6878178	6878165	**6877111**	**6877111**

Alpha band

Time-window	Null model	Model surprisal	Model predictor	Full model
300-500 ms	24072792	24072776	24069765	**24069741**
600-800 ms	24039177	24039112	24037364	**24037279**

Low-beta band

Time-window	Null model	Model surprisal	Model predictor	Full model
300-500 ms	29379683	29379645	29376655	**29376607**
600-800 ms	29352290	29352216	29348592	**29348505**
